# Thermopriming triggers splicing memory in Arabidopsis

**DOI:** 10.1093/jxb/ery062

**Published:** 2018-02-27

**Authors:** Yu Ling, Natalia Serrano, Ge Gao, Mohamed Atia, Morad Mokhtar, Yong H Woo, Jeremie Bazin, Alaguraj Veluchamy, Moussa Benhamed, Martin Crespi, Christoph Gehring, A S N Reddy, Magdy M Mahfouz

**Affiliations:** 1 Laboratory for Genome Engineering, Division of Biological Sciences, King Abdullah University of Science and Technology, Thuwal, Saudi Arabia; 2 Division of Biological Sciences, King Abdullah University of Science and Technology, Thuwal, Saudi Arabia; 3 Agricultural Genetic Engineering Research Institute (AGERI), Giza Egypt; 4 Institut des Sciences du Végétal (ISV), CNRS, UPR2355, Saclay Plant Sciences, Gif-sur-Yvette Cedex, France; 5 Department of Biology, Program in Molecular Plant Biology, Program in Cell and Molecular Biology, Colorado State University, Fort Collins, CO, USA

**Keywords:** Adaptation, alternative splicing, *Arabidopsis thaliana*, heat priming, heat responses, heat stress, stress memory

## Abstract

Abiotic and biotic stresses limit crop productivity. Exposure to a non-lethal stress, referred to as priming, can allow plants to survive subsequent and otherwise lethal conditions; the priming effect persists even after a prolonged stress-free period. However, the molecular mechanisms underlying priming are not fully understood. Here, we investigated the molecular basis of heat-shock memory and the role of priming in *Arabidopsis thaliana*. Comprehensive analysis of transcriptome-wide changes in gene expression and alternative splicing in primed and non-primed plants revealed that alternative splicing functions as a novel component of heat-shock memory. We show that priming of plants with a non-lethal heat stress results in de-repression of splicing after a second exposure to heat stress. By contrast, non-primed plants showed significant repression of splicing. These observations link ‘splicing memory’ to the ability of plants to survive subsequent and otherwise lethal heat stress. This newly discovered priming-induced splicing memory may represent a general feature of heat-stress responses in plants and other organisms as many of the key components are conserved among eukaryotes. Furthermore, this finding could facilitate the development of novel approaches to improve plant survival under extreme heat stress.

## Teaser

Thermopriming induces genome-wide differential gene expression and alternative splicing patterns, and establishes a ‘splicing memory’ that helps plants to survive subsequent and otherwise lethal heat stress.

## Introduction

Plants adapt to ever-changing environmental conditions by fine-tuning their molecular responses at the genome, epigenome, transcriptome, epitranscriptome, metabolome, and proteome levels to ensure survival and reproductive success (Hatfield and Prueger, 2015; Burgess *et al.*, 2016). Plants have molecular mechanisms to survive mild stress conditions, which constitute a basal stress-tolerance response. The level of basal stress tolerance varies across plant species and genotypes (Hilker *et al.*, 2016). Intriguingly, plants can acquire tolerance to lethal levels of stress through the establishment of ‘molecular stress memory’ of previous exposure to mild or severe transient stress (Sani *et al.*, 2013). Brief exposure to stress establishes a new cellular state that differs from the state of unexposed, naïve plants (Bruce *et al.*, 2007). Therefore, the responses (and thus tolerance) to future stress differ between primed and non-primed plants (Mauch-Mani *et al.*, 2017).

Stress memory allows primed plants to respond robustly and quickly to subsequent exposure to such stresses, which aids in their recovery (Prime-A-Plant Group, 2006; Hilker *et al.*, 2016). Priming could in future be applied in the field to make crops more stress resistant and productive (Liu and Charng, 2012), making the identification of factors controlling priming and stress-induced memory in plants an important focus of study. Priming-induced stress memory functions somatically within a generation; whether such a memory is transgenerational remains largely unknown.

Priming and the establishment of stress memory can help plants to survive a variety of abiotic stress conditions, including drought, heat, and salt, as well as biotic stress (Conrath, 2011; Baurle, 2016). The maintenance of acquired thermotolerance is crucial for successful priming and tolerance to subsequent exposure to otherwise lethal temperatures (Ikeda *et al.*, 2011). The acquisition and maintenance of thermotolerance are two separate processes (Jaskiewicz *et al.*, 2011; Lämke *et al.*, 2016*a*); the maintenance of thermotolerance is referred to as priming-mediated heat-stress memory (Baurle, 2016).

Due to global warming, heat stress poses an increasingly serious threat to plant survival and productivity, and therefore to agriculture and hence food security (Bita and Gerats, 2013). At the molecular level, increased temperature causes protein denaturation or misfolding, thereby compromising cellular viability (Morimoto and Santoro, 1998; Mittler *et al.*, 2012). Heat-stress responses are conserved across eukaryotic species including plants, animals, and fungi, and include the activation of heat-shock factors (HSFs) and the subsequent synthesis of heat-shock proteins (HSPs) (Swindell *et al.*, 2007; Liu *et al.*, 2009, 2011; Richter *et al.*, 2010; Nishizawa-Yokoi *et al.*, 2011). HSFs function as transcriptional activators of HSPs, and some HSPs positively regulate HSFs (Scharf *et al.*, 2012). HSPs function as molecular chaperones to protect the proteome against heat stress (Zhang *et al.*, 2010; Wu *et al.*, 2013; Weng *et al.*, 2014; Tang *et al.*, 2016). HSPs are grouped into families based on their molecular size, including HSP70, HSP90, and HSP101, as well as families of small HSPs (Larkindale and Vierling, 2008; Haslbeck and Vierling, 2015). Most plant genomes encode more than 20 HSFs with distinct functions, whereas animals and yeast genomes encode just a few.

The *Arabidopsis thaliana* genome contains more than 20 *HSF* genes, including eight whose roles in heat stress responses have been confirmed. One of them, *HSFA2*, contributes to the establishment of heat stress memory in primed plants (Charng *et al.*, 2007; Banti *et al.*, 2010). HSFA2 activates the chaperone-like protein HEAT STRESS ASSOCIATED 32 (HSA32), which may help maintain cellular homeostasis at high temperature (Charng *et al.*, 2006; Lin *et al.*, 2014). Other genes with a role in the somatic heat stress memory include *HSP70* and *HSP101*, which are up-regulated in response to heat shock (Stief *et al.*, 2014). HSA32 is required for the maintenance of heat stress memory, and therefore it is important to investigate how this protein differentially regulates the targets involved in its acquisition and maintenance (Baurle, 2016).

The molecular mechanisms of stress memory remain unclear, but different categories of stress-inducible genes may play different roles in memory. Heat stress-inducible genes are categorized into three groups. The activation of the first group is not sustained after relief of the stress, and these genes are not thought to play a major role in conferring heat stress memory. For example, expression of the heat stress-inducible genes *HSP70* and *HSP101* is induced by heat stress but subsequently declines after relief of the stress (Zhang *et al.*, 2010; Wu *et al.*, 2013; Lin *et al.*, 2014; Weng *et al.*, 2014). The activation of the second group is sustained after relief of the stress; these genes play a major role in heat stress-mediated memory (Stief *et al.*, 2014). For example, some HSP genes are induced during the heat stress memory phase, including *HSP21*, *HSP22.0*, and *HSP18.2* (Sedaghatmehr *et al.*, 2016). The activation of the third group is not sustained after stress relief, but these genes are readily activated by heat stress, and their expression is activated at higher levels after the second exposure to stress conditions (Lämke *et al.*, 2016*b*). Efforts have focused on identifying the genes that establish and maintain heat stress memory based on their enrichment after the relief of the stress. The levels and duration of such induction vary across plant species and genotypes.

The interplay between transcriptional and post-transcriptional regulation of gene expression is mediated at different levels and epigenetic factors are thought to play a role in establishing heat stress memory (Avramova, 2015; Hu *et al.*, 2015; Brzezinka *et al.*, 2016). Although many studies have focused on transcriptional regulation of stress responses, post-transcriptional responses may also play an important role. Alternative splicing (AS) is a post-transcriptional regulatory mechanism involving the processing of precursor mRNA (pre-mRNA) through the joining of exons in different patterns, resulting in the production of different mRNAs and thus different protein isoforms (Wang *et al.*, 2015). Alternative splicing enriches transcriptome and proteome diversity and also regulates gene expression through multiple mechanisms (Chen and Manley, 2009). Alternative splicing is widespread in photosynthetic eukaryotes, with nearly 61% of intron-containing genes in Arabidopsis exhibiting AS isoforms (Reddy *et al.*, 2013; Laloum *et al.*, 2018). Several types of AS events play key roles in gene regulation, representing an important post-transcriptional regulatory mechanism in response to different environmental conditions (Filichkin *et al.*, 2015). The constitutive pre-mRNA splicing of some transcripts is blocked in human and *Drosophila* cells under heat stress (Bond, 1988; Biamonti and Caceres, 2009; Shalgi *et al.*, 2014).

MostAS in plants involves intron retention (IR) (Iida *et al.*, 2004; Filichkin *et al.*, 2010); therefore, AS could also be used as a regulatory mechanism to monitor functional versus non-functional isoforms and to ensure the production of the correct isoforms at the right levels and times. Specifically, under stress conditions, IR could occur transiently to allow the plant to produce high levels of mRNA upon stress relief (Ding *et al.*, 2014; AlShareef *et al.*, 2017; Ling *et al.*, 2017). Early reports have indicated that RNA splicing is inhibited during heat-shock responses (Chang *et al.*, 2014), for example the splicing factor SRp38 mediates splicing repression in response to heat shock (Shalgi *et al.*, 2014). How heat stress affects constitutive and alternative splicing is currently unclear, and it remains to be determined whether heat stress can alter the patterns ofAS and produce certain pre-mRNA isoforms for proper responses to lethal temperatures. In other eukaryotes, including *Drosophila* and mice, *HSF1* and *HSF2* produce different AS isoforms and exhibit tissue-specific and temperature-responsive expression patterns (Lal *et al.*, 2015). Different thermosensitive AS responses have been reported in *Neurospora* (Diernfellner *et al.*, 2005). Heat stress-inducible AS isoforms of *HSFA2* in Arabidopsis may regulate heat-stress responses (Sugio *et al.*, 2009). One of the splice isoforms of HSFA2 has been shown to be important for autoregulation of *HSFA2* expression (Liu *et al.*, 2013). In Arabidopsis, heat stress-induced AS of the precursor of miR400 resulted in the accumulation of primary transcripts and reduced levels of mature transcripts (Yan *et al.*, 2012). A genome-wide analysis of heat stress-induced AS in *Physcomitrella patens* indicated that AS plays an important regulatory role in moss stress responses (Chang *et al.*, 2014).

Previous studies have focused on quantitative changes in gene expression in response to heat stress after the establishment of priming-induced heat stress memory (Charng *et al.*, 2007; Brzezinka *et al.*, 2016; Lämke *et al.*, 2016*b*). Because AS of pre-mRNA enhances transcriptome and proteome diversity in response to a variety of internal and external cues, including heat stress, we reasoned that AS might contribute to the co-ordination and regulation of gene expression in response to high temperature. Heat-responsive genes exhibit altered AS patterns, prompting us to investigate whether AS co-ordinates the regulation of gene expression during heat priming to establish heat stress memory. Therefore, we proposed that AS may contribute to heat stress-induced memory and we tested this hypothesis using our newly developed heat acclimation protocol, in which heat stress-induced priming leads to memory establishment that allows plants to survive a subsequent and otherwise lethal heat shock. In particular, we also investigated the genome-wide differential gene expression and AS patterns that mediate the establishment of priming and memory, and identified a group of genes with sustained activation levels that are implicated in the establishment of heat stress-induced memory in order to establish a link between AS and heat stress-induced memory.

## Methods

### Plant material and growth conditions

Seeds of wild-type *Arabidopsis thaliana* Col-0 were surface-sterilized with 10% bleach for 10 min and incubated at 4 °C for 2 d. The seeds were plated on half-Murashige and Skoog (MS) medium agar plates supplemented with 1% sucrose and incubated in a growth chamber (Model CU36-L5, Percival Scientific, Perry, IA, USA) under a 16-h photoperiod (white light, ~75 μmol m^−2^ s^−1^) and 8-h dark conditions at 22 °C for germination and seedling growth.

### Heat priming and heat-shock experiment

The heat-priming platform was designed and developed based on previous studies (Larkindale and Vierling, 2008). Twelve-day-old seedlings were divided into two sets. Set 1 was exposed to a four-step temperature-changing environment as follows. (1) Heat acclimation phase: temperature steadily increased from 22 to 45 °C within 6 h and held constant at 45 °C for 1.5 h. (2) Recovery/Memory phase: 4 d under normal growth conditions (16/8 h light/dark, ~75 μmol m^−2^ s^−1^, at 22 °C). (3) Heat-shock phase: temperature sharply increased from 22 to 45 °C and held constant at 45 °C for 1.5 h. (4) Recovery/Memory phase: normal growth conditions. Seedlings in Set 2 were grown under normal conditions for an additional 4 d (i.e. to 16-d-old), followed by exposure to the conditions described in steps (3) and step (4). Plant samples were collected at different time-points as specified below.

### RNA extraction and RNA-seq

Total RNA was isolated from the plants using an Oligotex mRNA Midi Kit (70042, Qiagen Inc., Valencia, CA, USA). The RNA-seq libraries were constructed using an Illumina Whole Transcriptome Analysis Kit following the standard protocol (Illumina, HiSeq system) and sequenced on the HiSeq platform to generate high-quality paired-end reads.

### RNA-seq data analysis and gene functional classification

Two replicate samples per time-point were used, except for time-point 3, where only one sample was used for analysis. The annotated Arabidopsis gene models were downloaded from TAIR10 (https://www.arabidopsis.org/). TopHat (Version 2.0.10) was used for alignment and to predict splice junctions (Trapnell *et al.*, 2009). Gene expression levels (fragments per kilobase of transcript per million mapped reads, FPKM) were calculated using Cufflinks (Version 2.0.0). The differentially expressed genes (DEGs) were identified using Cufflinks and the limma package in R (www.r-project.org). Very strict criteria were used to define DEGs: they had to simultaneously show more than 1.8-fold up-/down-regulation in both replicates, and *P*-values calculated by limma had to be less than 0.05. To filter out false-positive junctions, strict criteria (i.e. an overhang size of more than 20 bp and at least two reads spanning the junctions) were set as cut-off values (Cui *et al.*, 2014). JuncBASE was used to annotate all AS events based on the input genome co-ordinates of all annotated exons and all confidently identified splice junctions (Brooks *et al.*, 2011). Fisher’s Exact Tests were used to identify differential representation of each type of AS event. For all types of AS events, those with a *P*-value <0.001 were identified as significantly different. For IR, Fisher’s Exact Tests were performed on the intron-read counts and corresponding exon-read counts between different samples. In addition, IR events uniquely identified in any sample were considered significant only if there was at least five-fold coverage of support and the *P*-values of these events were assigned to zero. For alternative 5′-splice sites (SSs), 3′-SSs, and exon-skipping events, Fisher’s Exact Tests were performed on the comparisons of junction-read counts and the corresponding exon-read counts between samples. Gene ontology (GO) classifications were performed with the DAVID software (https://david.ncifcrf.gov/, (Dennis *et al.*, 2003). GO network analysis was performed with Exploratory Gene Association Networks (EGAN, http://akt.ucsf.edu/EGAN/).

### RT-PCR and RT-qPCR

Reverse-transcription PCR (RT-PCR) and reverse-transcription quantitative PCR (RT-qPCR) were performed as previously described (Ling *et al.*, 2017). Briefly, for RT-qPCR, digestion of contaminating DNA in total RNA samples was performed after RNA extraction using a RNase-Free DNase Set (Invitrogen, cat. no. 18068-015) following the manufacturer’s protocol. The total RNA was reverse-transcribed using a SuperScript First-Strand Synthesis System for RT-qPCR (Invitrogen) to generate cDNA. The qPCR was performed using Power SYBR Green PCR Master Mix (Invitrogen, cat. no. 4367659) under the following conditions: 95 °C for 10 min, followed by 40 cycles of 95 °C for 15 s, 60 °C for 1 min.

## Results

### Establishment of a heat stress-induced priming platform in Arabidopsis

**Fig. 1.**
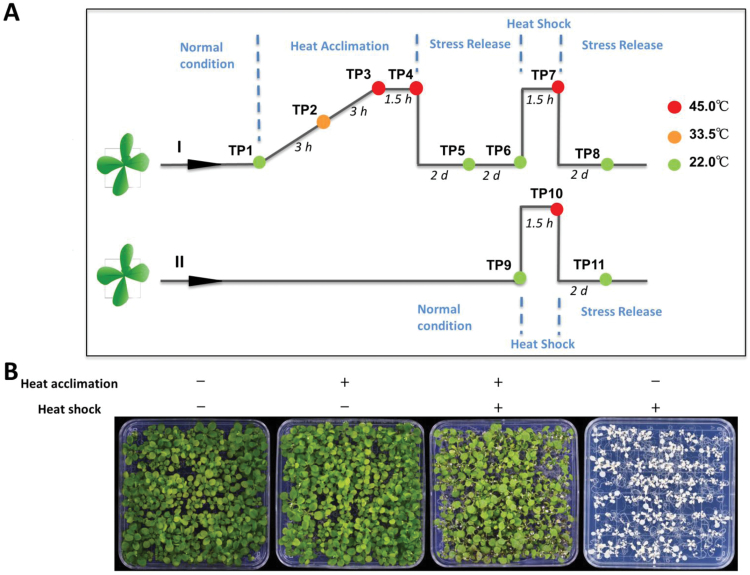
Outline of the platform used to induce heat-stress- priming. (A) Seedlings grown under long days were either primed (I) or not primed (II) with heat stress. During priming, the temperature was increased uniformly from 22 to 45 °C over 6 h, sustained at 45 °C for 1.5 h, and then brought down sharply to 22 °C. For the heat-shock process, the temperature was rapidly increased from 22 to 45 °C and then dropped down to 22 °C sharply after 1.5 h. The temperature of each sample collection time-point (TP) and the interval between the TPs are indicated. (B) Phenotypes of seedlings after different treatments. Pictures were taken when plants were 24 d old, 6 d after the heat shock.

To investigate the transcriptional and post-transcriptional regulation of heat-stress priming and memory, we developed a comprehensive heat stress-induced priming platform in Arabidopsis (see Fig. 1A). Briefly, we grew Arabidopsis seedlings for 12 d and collected some plants at the 4-leaf stage before exposure to heat stress (time-point 1, TP1). Subsequently, we initiated priming by exposing one set of plants (Set 1) to a gradual increase in temperature from 22 to 45 °C over the course of 6 h. We collected one sample after 3 h when the temperature reached 33.5 °C (TP2) and one sample after 6 h when the temperature reached 45 °C (TP3). These samples were used to study early responses to mild and severe heat stress, respectively. The remaining plants were incubated at 45 °C for 1.5 h, at which point another sample was taken (TP4). The remaining plants were transferred to 22 °C and a further two samples were collected, one after 2 d (TP5) and one after 4 d (TP6), prior to second exposure to high temperature (45 °C). The first exposure to heat was intended to induce priming, whereas samples TP5 and TP6 were used to investigate the recovery phase and the maintenance of somatic memory.

Subsequently, Set I (primed) and Set II (non-primed) were used for the second heat shock to test the effects of priming on the acquisition and maintenance of thermotolerance. We collected one sample from set II before exposure to heat shock (TP9) to serve as a control for TP6 plants, in which the expression of memory genes in response to heat stress would be maintained. The two sets were exposed to high temperature for 1.5 h and two samples were collected from Set I (TP7) and Set II (TP10). The plants were then maintained at 22 °C for 2 d, after which we collected samples from Set I (TP8) and Set II (TP11). These two samples were used to investigate the differential responses of primed versus non-primed plants. Plants were photographed and analysed phenotypically 6–10 d after treatment. Set I plants (primed for stress tolerance by an initial and gradual exposure to heat stress) performed much better than Set II plants (non-primed plants; Fig. 1B); this experiment was repeated more than three times with similar results. This priming approach involved different phases, including a priming phase, a recovery from priming phase, a second exposure phase, and a recovery phase after exposure to high temperature.

The second exposure to 45 °C constituted severe heat shock in Arabidopsis. However, primed plants were well adapted to 45 °C after the establishment of stress memory by the priming regime. Thus, our priming platform allowed the acquisition and maintenance of thermotolerance to be investigated at the genome scale, including the transcriptional and post-transcriptional levels of genes that were differentially regulated in their expression and/or splicing patterns to establish and maintain heat stress memory and thermotolerance in primed plants.

### Differential gene expression during different phases of heat-stress priming

After establishing the priming method, we investigated the genome-wide transcriptional changes induced by priming and their role in the establishment and maintenance of heat stress memory and priming. As described above, we collected samples at different time-points before, during, and after priming, recovery, and a second exposure to high temperature, and we isolated RNA and performed RNA-sequencing (RNA-seq) to investigate the role of transcriptional changes in inducing priming and maintaining heat stress memory and subsequently conferring thermotolerance.

Our comprehensive analysis of transcriptomes allowed us to identify differential gene expression patterns at all the different time-points. For example, to identify genes that function in the early response to high temperature, we compared the control TP1 plants with TP2 plants. To identify heat stress-response genes that function during severe conditions, we compared TP1 versus TP3 and TP4 plants. To identify all heat-responsive genes, we compared TP1 plants with TP2, TP3, and TP4 plants. The list of genes that were responsive to heat stress in our study was compared with publicly available microarray data for heat and abiotic stress-responsive genes using Genevestigator (https://genevestigator.com/gv/). The data were consistent with prior results, as shown in the heat map in Supplementary Fig. S1 at *JXB* online. Furthermore, GO analysis showed that these differentially expressed genes (DEGs) are involved in responses to heat and other abiotic stresses.

### Identification of differentially regulated genes potentially controlling heat-stress memory

Upon exposure to high temperature, thousands of genes were up- or down-regulated, as observed by comparisons between TP1 and TP2, and between TP3 and TP4. However, during the recovery phase after heat stress, the DEGs that maintained the same enrichment patterns were likely to function in maintaining heat stress memory. To identify such genes, we compared TP6 plants (4 d after recovery from heat stress) to TP9 (plants not primed and not exposed to heat stress). DEGs in TP6 plants should include genes whose expression is up- or down-regulated during heat stress and maintained after the recovery phase. Such regulation should be unique to primed plants and not be present in non-primed plants. Therefore, we identified genes whose expression changed by at least 1.8-fold after recovery from heat stress in priming.

**Fig. 2.**
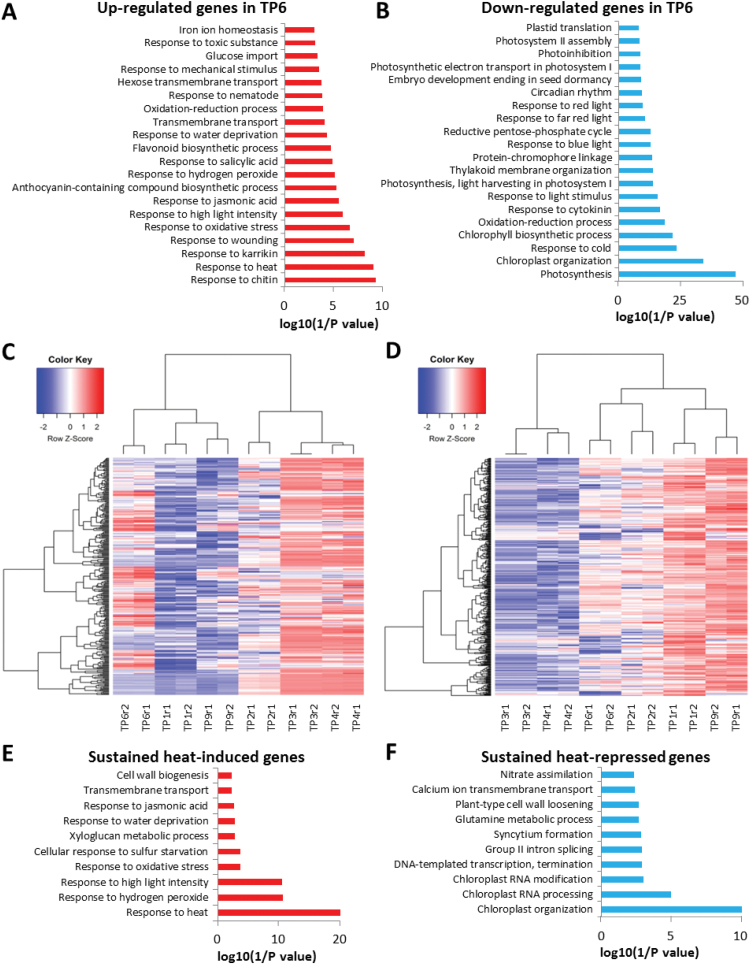
Differentially expressed genes between primed plants (time-point 6, TP6) and non-primed plants (TP9) before heat shock. Top 20 terms for (A) enrichment of biological processes in up-regulated genes and (B) in down-regulated genes at TP6 compared to TP9. (C, D) Heatmaps indicating that the heat-memory genes are still actively up- or down-regulated 4 d after heat acclimation: (C) up-regulated genes, (D) down-regulated genes. Top 10 terms for enrichment of biological processes in (E) up-regulated memory genes and (F) in down-regulated memory genes. TP1, control (22 °C); TP2, priming phase (33.5 °C); TP3, priming phase (45 °C); TP4, priming phase (45 °C); TP6, 4 d after heat priming (22 °C); and TP9, control for TP6 (22 °C). See Fig. 1 for the temperature regime.

Approximately 2600 genes were differentially regulated at TP6 compared to TP9, including 982 up-regulated genes and 1642 down-regulated genes. This list of genes should include those that control the acquisition and/or maintenance of heat stress memory, thereby allowing primed plants to survive subsequent exposure to high temperature. GO analysis indicated that the up-regulated genes are involved in biological processes including responses to heat, high-light stress, water deprivation, and other abiotic/biotic stresses, whereas the down-regulated genes are involved in photosynthesis, chloroplast organization, chlorophyll biosynthetic process, and light stimulus (Fig. 2A, B).

It is possible that some DEGs between TP6 and TP9 may not be related to the establishment of heat stress memory. To address this, we determined the overlap between heat-responsive genes identified during the priming phase (TP1 versus TP2, TP3, and TP4) and the putative heat stress memory genes identified above by comparing TP6 with TP9 plants. Using the heatmap.2 function in R, we found that TP6, TP1, and TP9 DEGs clustered together, as did TP3 and TP4 (Fig. 2C, D). To determine whether such DEGs in TP6 are associated with the heat stress response, we used Genevestigator to compare our data with the publicly available microarray data. This analysis revealed that DEGs during the recovery phase were associated with the heat stress response (see Supplementary Fig. S2). We also performed GO analysis of these DEGs, which potentially control or help maintain heat stress-induced memory (Fig. 2E, F). Our data showed that ‘response to heat’, ‘response to hydrogen peroxide’, and ‘response to high light intensity’ were the most enriched terms in up-regulated genes, whereas ‘chloroplast organization’, ‘chloroplast RNA processing’, and ‘chloroplast RNA modification’ were the most enriched terms in down-regulated genes. Finally, we used EGAN to construct gene networks of heat-responsive genes (Supplementary Fig. S2), and the results showed that a group of memory genes are heat and abiotic stress-responsive. Overall, these results showed that some important heat-responsive genes maintained their expression patterns during the 4-d post-priming phase, which would help the primed plants sustain subsequent heat shock exposure.

### Heat stress-primed versus non-primed plants respond differently to a second exposure to high temperature

**Fig. 3.**
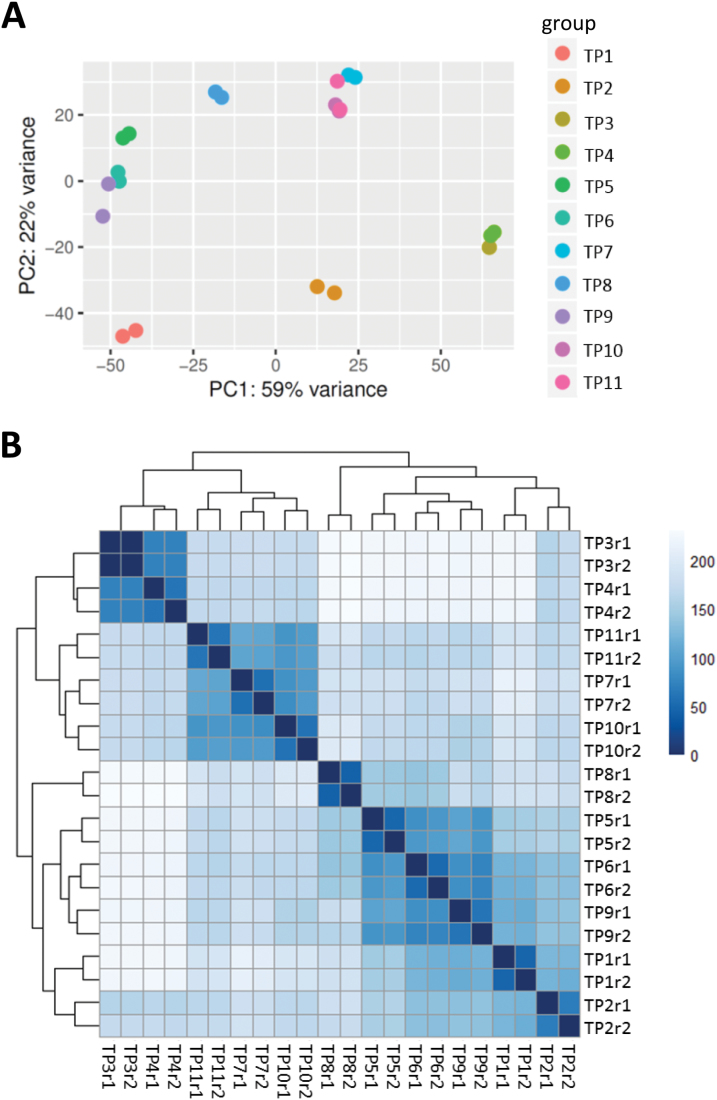
Clustering of gene expression patterns between different samples. (A) Clustering and relationships between conditions by principal component analysis (PCA) using a dimensionality reduction strategy in cummeRbund (http://compbio.mit.edu/cummeRbund/). (B) Distance matrix among all samples using read counts normalized using the rlog function of DESeq2 (doi:10.18129/B9.bioc.DESeq2). The distance matrix shows that time-points TP1, TP2, TP5, TP6, TP8, and TP9 grouped together, and that TP3, TP4, TP7, TP10, and TP11 grouped together. See Fig. 1 for the temperature regime.

Next, we investigated why primed plants survived the second exposure to heat stress better than non-primed plants. We initially clustered the gene expression profiles using two methods: principal component analysis (PCA) and distance matrix (Fig. 3A, B). The TP1, TP2, TP5, TP6, TP8, and TP9 samples clustered together, which was expected since these plants either were not exposed to heat stress, were exposed to only moderate heat stress, or had recovered from heat stress. Intriguingly, TP11 plants (non-primed plants exposed to heat stress) clustered with TP3, TP4, TP7, and TP10 plants, suggesting that the gene expression profile of TP11 is more like that of plants exposed to heat stress, even after a 2-d of recovery. This result suggests that although heat stress was relieved in the latter samples, the gene expression profiles of these plants mimicked those of plants under heat stress conditions. Equally intriguing, and in contrast to TP11 plants, the gene expression profile of TP8 plants was similar to those of non-primed control plants, suggesting that in primed plants gene expression patterns were reset back to normal once the stress was relieved, but those of non-primed plants were not reset. This would explain why the primed plants survived exposure to high temperature while the non-primed plants did not. There were thousands of DEGs found between TP8 and TP11. Furthermore, we compared these DEGs to known heat-responsive genes and found that they substantially overlapped (~50%), further confirming the high similarity of gene expression profiles between TP11 and plants under severe heat stress conditions. We generated heatmap profiles and they supported this conclusion (see Supplementary Fig. S3).

### Transcriptional regulation of a subset of genes may mediate heat stress-induced priming memory

**Fig. 4.**
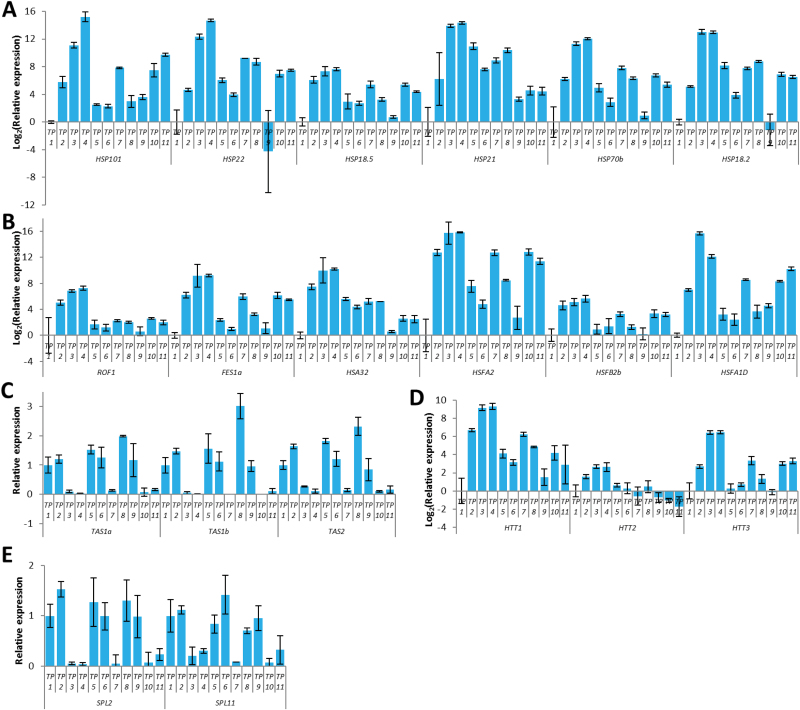
Expression of heat-responsive genes. (A) Heat-shock protein (HSP) genes show up-regulation by heat stress and priming, and most of them maintain higher expression levels for 4 d (time-point 6, TP6) than were observed before heat stress or priming (TP1 and TP9). (B) Heat-shock factors (HSFs) and heat-responsive marker genes show up-regulation by heat stress and priming. (C, D) *trans-acting siRNA precursors 1a* (*TAS1a*), *TAS1b*, and *TAS2* are down-regulated by heat stress (C), whereas their target genes, *HEAT-INDUCED TAS1 TARGET1* (*HTT1*), *HTT2*, and *HTT3* are induced during heat stress (D). (E) Targets of miR156, *SPL2*, and *SPL11* genes are down-regulated by heat stress. See Fig. 1 for the temperature regime. (This figure is available in colour at *JXB* online.)

Our RNA-seq data analysis revealed that a subset of transcriptionally regulated genes may mediate the acquisition and maintenance of heat stress memory. We validated the expression patterns of a subset of these genes using qRT-PCR. First, we validated the differential expression of a number of *HSP*s (*HSP101*, *HSP22*, *HSP18.5*, *HSP21*, *HSP70B*, and *HSP18.2*) and *HSF*s (*HSF32*, *HSFA2*, and *HSFB2b*) at all time-points. Our qRT-PCR data corroborated the RNA-seq data (Fig. 4A, B). Next, we validated expression of several other heat-responsive genes. The qPCR results demonstrated that many heat-responsive genes, including those that are involved in non-coding RNA-dependent pathways, were differentially regulated. The *TRANS-ACTING SIRNA PRECURSORS 1a* (*TAS1a*), *TAS1b*, and *TAS2* were down-regulated by heat stress, whereas their target genes, *HEAT-INDUCED TAS1 TARGET1* (*HTT1*), *HTT2*, and *HTT3* were induced during heat stress. Moreover, targets of miR156 (*SPL2* and *SPL11*) were down-regulated by heat stress (Fig. 4C–E).

### Alternative splicing regulates plant responses to heat stress and contributes to priming

Several reports have implicated AS in regulating plant responses to biotic and abiotic stress, prompting us to investigate whether it is involved in the acquisition and/or maintenance of heat stress memory. To analyse changes in pre-mRNA splicing during the priming or recovery phase, or upon exposure to the second heat stress phase, we employed our recently developed pipeline (Cui *et al.*, 2014) and performed three steps: prediction of splice junctions, filtering of false-positive junctions, and annotation of AS events. RNA-seq reads were mapped onto the Arabidopsis genome (TAIR 10) to predict the splice junctions using the TopHat software, which identifies the splice junctions of exonic and intronic sequences (Trapnell *et al.*, 2009). Our alignment generated 148 701 splice junctions from the 21 libraries at the 11 time-points for the Set I and Set II samples after removing the false-positives containing short overhangs and low coverage, as previously described (Cui *et al.*, 2014). When we compared these junctions with gene annotations from TAIR 10, we found that 70% of were previously annotated and 30% were novel junctions. These data sets were used for comparisons with annotated genes to identify all AS events at all time-points from Set I and Set II, including 127 555 IR events, 6574 alternative 5′-SS events, 19 577 alternative 3′-SS events, 19 mutually exclusive exon events, and 956 co-ordinate cassette exon events (see Supplementary Table S1). Of the IR events, 38 131 had at least five reads supporting the event and >80% of them were found at all time-points. Furthermore, our data indicated that 53% of the intron-containing genes exhibited AS under normal conditions (TP1), 46% under mild stress (TP2), 46% under severe stress (TP4), 55% under lethal stress (TP10), 50% at the recovery phase (TP6), and 52% upon a second exposure to lethal stress (TP7, Supplementary Table S2). Our results indicated that IR was the prevalent form of AS under heat stress compared to other forms, including alternative 5′-SS and 3′-SS (Supplementary Table 1).

**Fig. 5.**
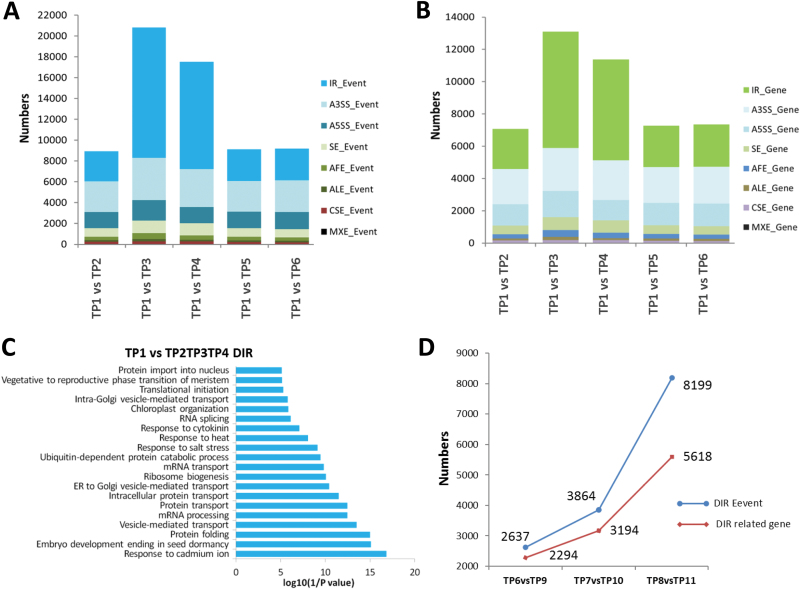
Differentially expressed alternative splicing (AS) events and related genes between primed plants and their respective controls (see Fig. 1 for the temperature regime). (A, B) Differential expression of AS events (A) and related genes (B) between control (time-point 1, TP1) and other plants. (C) Functional classification of heat-responsive genes with intron retention (IR). (D) Differential expressed IR events and related genes between primed and non-primed plants before heat shock, after heat shock, and after 2 d recovery.

We analysed the different AS events between control plants (TP1) and plants in the priming phase subjected to gradual mild (TP2) or severe heat stress (TP3 and TP4). We observed a significant increase in IR during the priming phase in plants subjected to severe heat stress in TP1 versus TP3, and in TP1 versus TP4 comparisons (Fig. 5A, B). However, during the recovery phase, the number of IR events and genes involved in these events significantly decreased, indicating that heat stress significantly repressed the splicing machinery, leading to the accumulation of pre-mRNAs with retained introns (Fig. 5A, B). GO analysis of the genes that underwent IR in response to heat revealed that this set of genes was enriched in biological process categories involving abiotic stress, including heat stress, RNA splicing, protein folding, and transport (Fig. 5C). These biological processes are modulated during heat episodes to help plants survive the transient stress. In addition, comparisons between primed and non-primed plants revealed that the number of genes with differentially retained introns increased in plants exposed to lethal heat stress. Surprisingly, this number increased more sharply in the recovery phase 2 d after exposure to lethal heat stress (Fig. 5D).

### IR contributes to the establishment of heat-stress memory

**Fig. 6.**
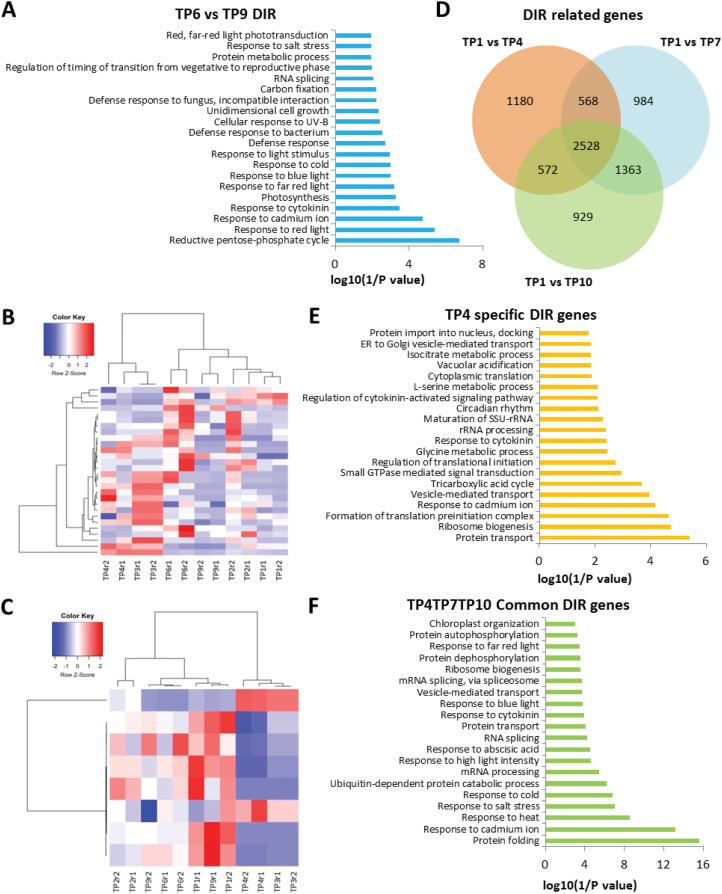
Different expressed intron retention (IR) events and related genes at various time-points (TPs). (A) Gene ontology (GO) analysis of differentially expressed IR genes for TP6 versus TP9. (B, C) Clustering of expression profiles of the genes defined as heat-memory alternative splicing events: (B) 28 genes with more retained introns in TP6, (C) eight genes with more retained introns in TP9. (D) Venn diagram showing a comparison of differential IR (DIR)-related genes between samples after heat priming (TP4) and heat shock (TP7 and TP10). (E) Functional categorization (biological process) of DIR-related genes in the heat-priming sample only (TP4). (F) Functional categorization (biological process) of DIR-related genes in all three samples (TP4, TP7, and TP10). The top 20 enriched pathways are shown. See Fig. 1 for the temperature regime.

Next, we investigated whether AS patterns were sustained and remained in plants during the recovery phase (memory establishment phase) and thereby whether they could mediate heat stress-induced memory. We compared the differential intron retention (DIR) events (which significantly increased under heat stress) of TP6 versus TP9 plants. Primed TP6 plants still maintained some DIR events. GO analysis of this group of genes indicated that they were associated with abiotic stresses, response to light stimulus, as well as RNA splicing (Fig. 6A). We then investigated whether the DIR events in TP6 were present in TP2, TP3, and TP4 in order to corroborate our hypothesis that such events are essential for the plant response to heat stress. Interestingly, our data revealed DIR events from a group of 36 genes (Fig. 6B, C, Supplementary Table S3).

To understand the effect of heat shock- and priming-induced changes on IR, we analysed the functional categories and cellular processes regulated by such genes. We identified more than 6000 genes with DIR events during heat shock (TP10) and 6242 genes during the priming phase (TP4), and 6192 genes during the second exposure phase (TP7), when compared to control plants (TP1). A subset of 1180 genes with DIR events was unique to the priming phase; some of them might be responsible for the establishment and maintenance of heat stress memory. Functional analysis of these genes using the DAVID software indicated that they are involved in biological processes including protein transport processes, ribosome biogenesis, rRNA processing, and regulation of translational initiation (Fig. 6E). Interestingly, the functional category ‘response to heat stress’ was markedly enriched among the DIR genes, and such genes were also enriched in the categories ‘heat stress responses’ and ‘memory establishment’, primarily after exposure to lethal temperatures (Fig. 6F).

### Differentially expressed and alternatively spliced genes are co-regulated in response to heat-stress priming

**Fig. 7.**
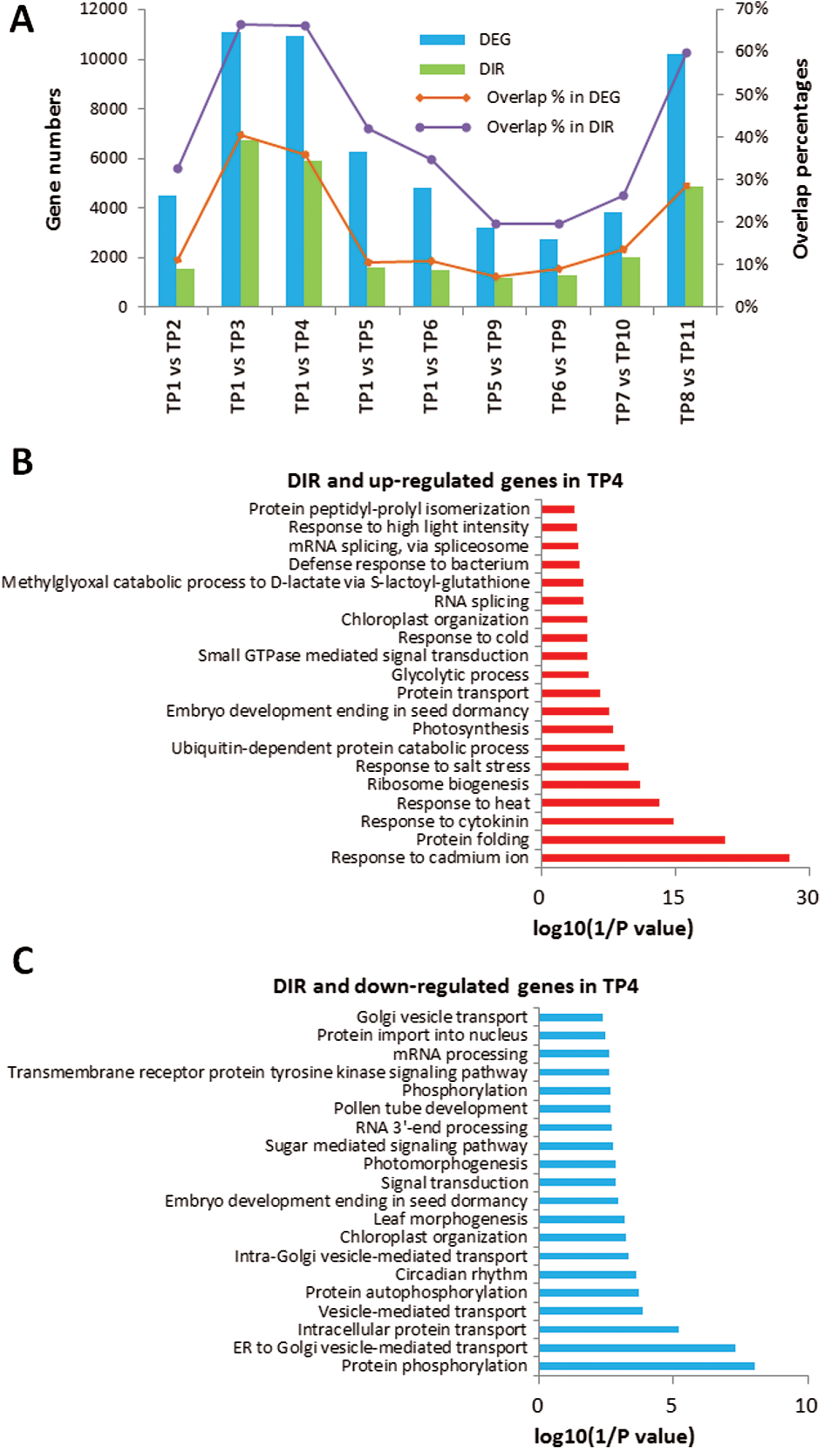
Differentially expressed genes (DEGs) and differential intron retention (DIR) genes between various time-points. (A) The number of DEGs, the number of DIRs, and the percentages of overlapping genes in DEGs and DIRs found in different comparisons of time-points (TPs). (B) Gene ontology (GO) analysis of up-regulated and with DIR genes between TP4 and TP1. (C) GO analysis of down-regulated and with DIR genes between TP4 and TP1. See Fig. 1 for the temperature regime. (This figure is available in colour at *JXB* online.)

Next, we investigated whether the DEGs during different phases of heat-stress priming (acquisition) and maintenance of heat-stress memory were co-regulated. Our RNA-seq analysis revealed that 4483, 11 105, 10 963, 6264, and 4842 genes were differentially regulated at TP2, TP3, TP4, TP5, and TP6, respectively, compared with the control (TP1), and 3233 and 2624 genes were differentially regulated at TP5 and TP6, respectively, compared with TP9 (Fig. 7A) (fold change >1.8 and *P*<0.05). Intriguingly, when we compared genes with DIR events with differentially regulated genes, we found significant overlap between DEGs and DIR genes during different phases. The number of overlapping genes increased when plants suffered from increasing temperature (from TP2 to TP3 and TP4), with more than 65% of DIR genes being DEGs. The number of overlapping genes then decreased during the recovery phase. Interestingly, the overlapping genes between primed and non-primed plants during the recovery phase after lethal heat shock sharply increased (TP8 vs TP11), suggesting that these two processes are, to a certain extent, co-regulated to respond to heat stress (Fig. 7A). It should be noted that differential expression of many genes may also be regulated by AS.

Nonetheless, many DIR genes were also DEGs that were co-regulated during the priming phase. GO analysis revealed that these genes, especially those that underwent IR in response to heat, are involved in abiotic stress responses, including heat stress, protein folding and transport, as well as regulation of mRNA processing (Fig. 7B, C). Interestingly, during the recovery and maintenance of the memory phase, we found 1264 genes with DIR events and 246 of these genes were differentially expressed, indicating that the transcriptional and post-transcriptional processes were co-regulated. To investigate whether these subsets of differential AS and DEGs were involved in heat-stress memory acquisition and maintenance, and thereby in facilitating plant survival upon subsequent exposure to heat stress, we examined their expression/splicing patterns during priming. Our data revealed that 112 of 246 genes are either up- or down-regulated at the transcriptional level and also showed DIR during priming (TP4) (see Supplementary Fig. S4).

### Heat-stress priming establishes splicing memory

Because our AS analysis revealed that responses to heat stress were regulated post-transcriptionally via effects on pre-mRNA splicing and that some AS patterns were associated with heat-stress memory, we asked whether AS contributes to the establishment of heat-stress memory and thereby helps plants survive their next exposure to stress. We compared the AS events in heat-stress primed (TP8) versus non-primed plants (TP11). Interestingly, the primed plants (TP8) were capable of efficient splicing and produced splicing patterns similar to those of control plants not exposed to high temperature. By contrast, the non-primed plants (TP11) exhibited high levels of IR and were therefore still producing splicing variants mimicking heat-stress conditions (see Supplementary Fig. S5). Thus, the primed plants appeared to maintain splicing memory, and after relief from the second exposure to stress they ‘remembered’ to undergo efficient splicing, to produce splicing patterns similar to those of control plants under non-stressful conditions in order to support growth and development.

To validate our RNA-seq data, we performed RT-PCR for several key genes to determine their splicing patterns in primed and non-primed plants. Our RT-PCR data corroborated our RNA-seq data, showing that non-primed plants were not capable of efficient splicing to produce patterns similar to those of control plants under non-stressful conditions, as evidenced by the higher levels of IR. By contrast, the primed plants were capable of producing similar splicing patterns to those of the control (see Supplementary Fig. S6).

**Fig. 8.**
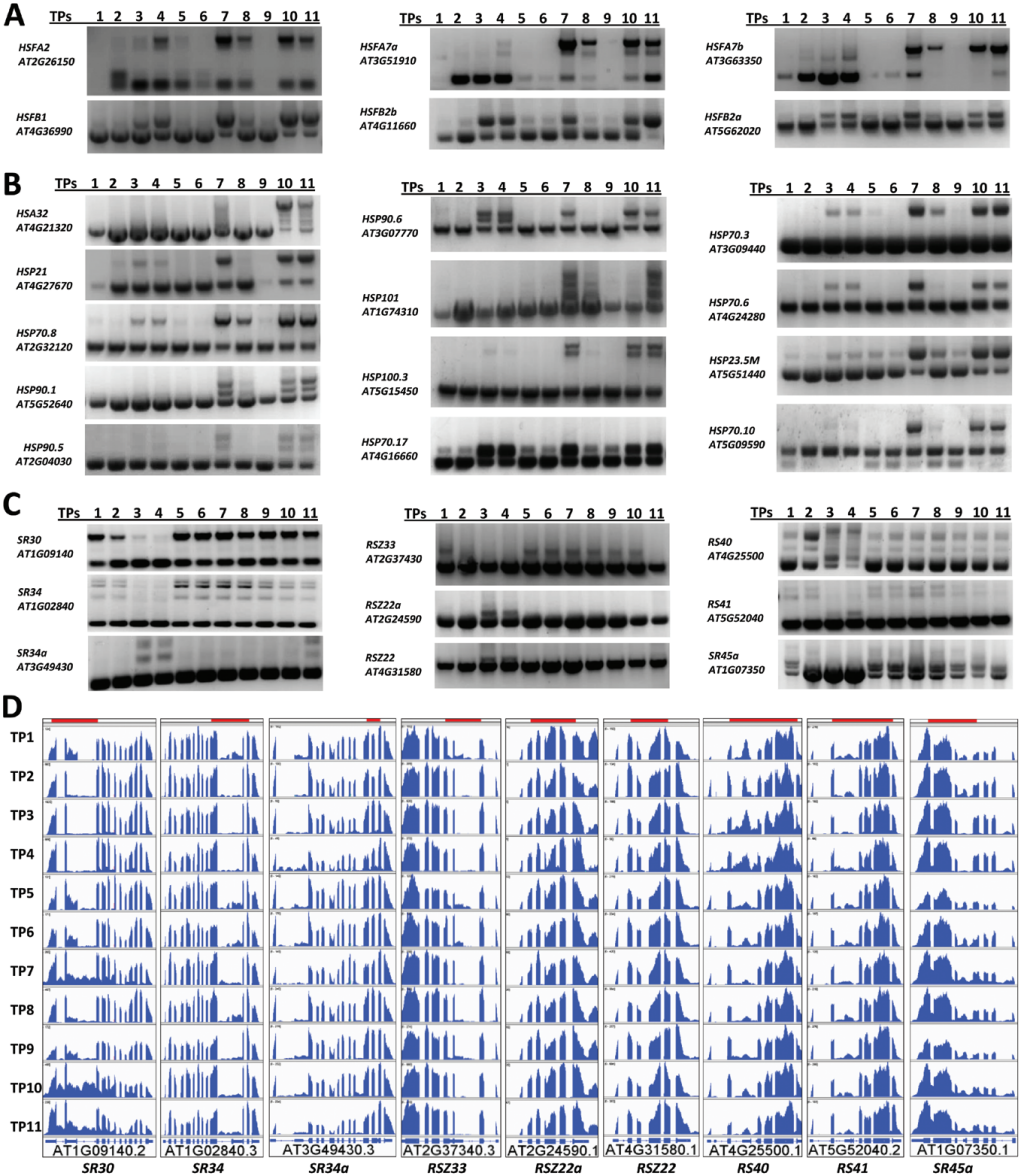
Differential splicing pattern of HSF genes, HSP genes and SR genes at different time-points. (A–C) RT-PCR was performed using primers that flank intron(s). (A) Splicing patterns of heat-shock factor (*HSF*) genes. *HSFA2*, *HSFA7a*, and *HSFA7b* expressed fewer intron-retained isoforms after heat priming (TP3 and TP4) when compared to after heat shock (TP7 and TP10). *HSFB1*, *HSFB2a*, and *HSFB2b* accumulated more intron-retained isoforms after heat priming when compared to after heat shock. (B) Splicing patterns of heat-shock protein (*HSP*) genes. genes show different ratios of intron retention after heat priming and heat shock. Most genes, except for *HSP90.6* and *HSP70.17*, expressed fewer intron-retained isoforms after heat priming (TP3 and TP4) when compared to after heat shock (TP7 and TP10). (C) Splicing patterns of a group of serine-/arginine-rich (*SR*) genes. Specific of isoforms of SR genes were induced in heat priming (TP3 and TP4). (D) Gene structure and intron retention of interesting regions from the nine *SR* genes in (C) as illustrated using the IGV program (http://software.broadinstitute.org/software/igv/). Coverage of RT-PCR-amplified fragments are indicated by the shaded bars at the top of each panel. (This figure is available in colour at *JXB* online.)

Heat priming differentially affects pre-mRNA splicing of *HSF*s. Members of HSF class B, namely HSFB1 and HSFB2b, repress heat-inducible genes, including *HSF*s under non-heat conditions and in the attenuating period (Ikeda *et al.*, 2011), whereas heat-induced transcript factors, class A HSFs, including HSFA2 and HSFA7a, play an important role in thermotolerance (Larkindale and Vierling, 2008; Meiri and Breiman, 2009). Interestingly, in our RT-PCR experiments *HSFB1*, *HSFB2a*, and *HSFB2b* showed a dramatic increase in IR in both the heat-priming and heat-shock phases, whereas splicing of *HSFA2*, *HSFA7a*, and *HSFA7b* was much less affected in the priming phase (Fig. 8A, Supplementary Fig. S7). Subsequently, although expression of Class A and B HSFs was up-regulated by heat priming/heat shock, plants had increased levels of class A but few functional class B transcripts in the priming phase, whereas the transcript levels of both classes of proteins were clearly affected by heat shock.

Heat priming induces specific isoforms of serine-/arginine-rich (SR) genes, which are the key regulators of AS, and probably modulates pre-mRNA splicing of HSPs. From our previous qPCR results (Fig. 4A, B) and RNA-seq data, we found that most *HSP*s and *HSF*s were highly induced by heat priming (TP3 and TP4) and heat shock (TP7 and TP10). We then tested whether the splicing patterns of *HSP* and *HSF* pre-mRNAs were altered during the heat-priming/heat-shock program. We found that pre-mRNA from a subset of *HSP* genes, including *HSP21*, *HSP101*, *HSP70.10*, *HSP70.6*, *HSP90.5*, and *HSP100.3*, were alternatively spliced, mostly due to IR, in response to heat stress/priming. Interestingly, the levels of intron-retained isoforms were much higher in the heat-shock compared to the heat-priming phase, except for *HSP70.17*. Moreover, *HSP70.10* expressed mostly the constitutively spliced isoform in the heat-priming phase, but produced two or more isoforms during other phases. By contrast, *HSP90.6* produced more isoforms in the priming phase, compared to the other phases (Fig. 8B).

In contrast to the HSPs, our RT-PCR analysis shows that pre-mRNAs encoding SR proteins tended to be alternatively spliced in the heat-priming process. In addition to retained intron splicing of transcripts, *SR30*, *SR45a*, *SR34*, and *RS41* underwent other AS events, including alternative first-exon and alternative last-exon events, which may have resulted in addition/deletion of a functional domain of proteins. However, those genes were affected less in splicing during heat shock when compared with the control conditions (Fig. 8C, D). Thus, it remains to be confirmed whether AS of *SR* pre-mRNAs agrees with what we found in *HSP*s genes, namely that those splicing processes were less affected during heat priming. Our RT-PCR data on splicing corroborated our RNA-seq data, and these results suggested that heat stress leads to splicing repression and significant levels of IR. Furthermore, heat priming and heat shock induced different levels of IR. During the memory establishment phase (TP5 and TP6), splicing returned to control-like levels. The second exposure to heat stress in primed plants (TP7) and the first exposure to heat stress in non-primed plants led to significant levels of IR, indicating splicing repression. However, after relief from the stress, the primed plants were capable of efficient splicing, unlike non-primed plants, which exhibited splicing repression mimicking stress conditions. These findings suggested that it might be possible that heat-stress priming could be established at the post-transcriptional level through the maintenance of splicing memory. Efficient splicing after heat-stress relief is crucial for plant survival and the completion of their life cycle.

## Discussion

Global climate change is having a substantial impact on agriculture worldwide, resulting in losses in crop productivity, and it poses a serious threat to global food security. Plants live in a dynamic environment with fluctuating temperature regimes, including both predictable and unpredictable seasonal changes. Prior exposure to mild heat stress primes plants, allowing them to acquire and maintain heat-stress memory, leading to increased thermotolerance. Therefore, priming can be viewed as a form of somatic stress memory, which is crucial for plant survival under subsequent stress conditions. Understanding the molecular mechanisms underpinning the acquisition and maintenance of heat stress is of paramount importance to both basic and applied research. Specifically, which factors control the acquisition and maintenance of heat-stress memory? How is this memory established, and how long does such somatic memory persist?

Priming conditions induce transcriptional changes that are sustained during the recovery or the memory-establishment phase (Ding *et al.*, 2012). Several key components function in heat stress-induced priming; however, no previous study has attempted to identify these factors at a genome-wide scale (González-Schain *et al.*, 2016). In this study, we established a comprehensive heat stress-induced priming platform that allowed us to investigate transcriptional and post-transcriptional regulation in response to priming and subsequent exposure to high temperature. Our platform included a priming phase, a stress-recovery and memory-establishment phase, and a stress-challenge phase. During the priming phase, we subjected plants to a gradual increase in temperature and collected samples after the temperature reached 33.5 °C. This sample (TP2) was used to identify the early-response genes and to determine whether the differential regulation of these genes was sustained during severe heat stress (TP3 and TP4). Our results indicated that genes responsive to heat and other abiotic stresses, including drought and cold, and hormones such as abscisic acid, were differentially regulated. Under severe heat stress, the transcriptional patterns involved up-regulation of *HSF* and *HSP* genes, as previously reported (Charng *et al.*, 2007; Mittler *et al.*, 2012).

Next, we analysed the priming and recovery phases in order to identify genes that underpin the acquisition and maintenance of heat-stress memory. Interestingly, clustering analysis of gene expression profiles showed that genes expressed during the recovery phase clustered with control samples (TP1 and TP9) compared to plants subjected to severe stress during the priming phase (TP3, TP4). These results indicated that in the recovery phase most gene expression profiles were reset to the expression patterns observed under normal conditions; some DEGs retained their transcriptional level in order to increase plant tolerance to subsequent exposure to heat stress; these DEGs constitute the memory genes.

Plants acquire thermotolerance through the activation of HSPs, the central proteins that confer tolerance to high temperature (Larkindale and Vierling, 2008). We identified genes encoding HSFs and HSPs among the genes controlling heat-stress memory. For example, *HSP21* was induced and its expression maintained during the recovery phase, indicating that it plays a role in heat stress-induced memory. The accumulation of HSP21 was recently shown to increase the thermomemory capacity of Arabidopsis, thereby increasing thermotolerance (Sedaghatmehr *et al.*, 2016). Likewise, HSFA2 is required for the maintenance of heat stress-induced memory, and we found that *HSFA2* was still induced at the end of the recovery phase (TP6) (Lämke *et al.*, 2016*a*, 2016*b*). Furthermore, the small Arabidopsis HSP gene *At-HSP17.6A* is regulated by heat, osmotic, and drought stress, and plants overexpressing this gene exhibit enhanced stress tolerance (Sun *et al.*, 2001). Moreover, the plant-specific heat-response protein gene *HSA32* was up-regulated and maintained in primed plants (TP6), indicating its involvement in the maintenance of heat-stress memory. This result corroborates the finding that HSA32 is required for the maintenance of acquired thermotolerance after a long recovery following acclimation treatment (Charng *et al.*, 2006). Heat-stress memory is established during the first exposure to high temperature.

Epigenetic factors help control priming responses and help maintain priming memory (Berry and Dean, 2015), and epigenetic regulation would ultimately affect the transcriptional patterns of genes. Therefore, the expression patterns identified in this study could have involved epigenetic regulation. Such patterns must be maintained to help confer thermotolerance to heat stress. For example, sustained H3 Lysine 4 tri-methylation and demethylation persist, even after active transcription of these loci has dropped to normal levels (Lämke *et al.*, 2016*a*). Therefore, it is possible that such loci are epigenetically marked for hyper-induction upon recurring exposure to heat stress. Indeed, when we compared primed and non-primed plants subjected to a second exposure to heat stress, we identified genes that were hyper-induced in the primed plants. However, these genes did not sustain high transcript levels during the recovery phase of memory establishment and maintenance after the first exposure to heat stress. Therefore, such genes could be epigenetically marked for hyper-induction under recurring heat stress.

Alternative splicing is a key mechanism that eukaryotes use to produce diverse transcriptomes and proteomes to support cellular functions, including during abiotic stress responses (Chen and Manley, 2009; Reddy *et al.*, 2013). The role of AS in establishing priming memory, recovery, and resetting is largely unexplored. AS patterns ensure differential regulation of protein abundance to allow plants to overcome heat stress and complete their life cycle. The targeted production of specific transcript isoforms may underpin priming memory and the resetting of such memory to allow an organism to develop thermotolerance. Splicing repression under heat-stress conditions has been observed in multiple eukaryotic systems and might, therefore, represent a conserved mechanism across eukaryotic species (Chang *et al.*, 2014). Nuclear retention of unspliced transcripts under heat stress may prevent the production of aberrant proteins or peptides, thereby reducing the molecular burden on chaperones and the proteasome machinery (Reddy *et al.*, 2013; Staiger and Brown, 2013). Recent studies indicate that IR has an important role in fine-tuning gene expression depending on the physiological or developmental status of cells or organisms (Boothby *et al.*, 2013; Braunschweig *et al.*, 2014). The unspliced transcripts may represent a cellular reservoir that is ready for splicing upon the relief of heat-stress conditions. In support of this, studies using a fern system showed that partially spliced transcripts are stored in nuclear speckles in association with an exon-junction complex protein and splicing of these stored intron-containing mRNAs is triggered by spermatogenesis during specific developmental stages (Boothby and Wolniak, 2011; Boothby *et al.*, 2013). The subset of genes whose splicing patterns were unaffected by heat stress could result from increased stability of pre-made and processed mRNA or from the maintenance of an efficient splicing process. Perhaps the splicing of these unaffected genes occurs in molecular bodies enriched with splicing factors to ensure efficient splicing under heat stress (Ali *et al.*, 2003). These unaffected genes were enriched in the molecular-function categories pertaining protein folding, which are commonly induced under heat-stress conditions.

We investigated whether the repression of splicing was indiscriminate or whether subsets of genes were differentially spliced under high-temperature conditions. Because IR is the predominant form of AS in plants, we investigated the patterns of IR throughout our priming platform. Specifically, we investigated whether primed plants would maintain IR during the recovery and memory-establishment phases and whether these primed plants would respond differently after the second exposure to heat stress at the AS level. We found that plants responded to heat stress by accumulating significant levels of IR events, indicating splicing repression. During the recovery phase, these IR events declined to normal levels. However, after the second exposure to heat stress, the response of primed plants was drastically different from that of non-primed plants, which accumulated higher levels of IR products, indicating significant splicing repression. Intriguingly, the primed plants did not respond to the second exposure to heat stress by accumulating high levels of IR; these primed plants ‘remembered’ to undergo splicing and produced splicing patterns similar to those of control plants under normal conditions. Therefore, primed plants can maintain a ‘splicing memory’, whereby after relief of the stress they exhibit efficient splicing and splicing patterns that support plant growth and development. This splicing memory is a key factor conferring thermotolerance to primed plants. We validated the splicing patterns of several genes from primed and non-primed plants after exposure to lethal heat stress. Our data corroborated the RNA-seq data, showing significant IR retention after the exposure to lethal heat-stress in non-primed plants, whereas primed plants exhibited efficient splicing to support plant growth and development and therefore survived the exposure to lethal heat stress.

This is the first report demonstrating that heat-stress priming results in splicing memory, which allows the primed plants to execute efficient splicing upon relief of the stress. Transcripts of key genes regulating plant responses to heat stress were alternatively spliced, and these AS patterns were different between primed and non-primed plants. Moreover, the expression of specific subsets of genes controlling key processes of pre-mRNA splicing, including genes encoding splicing-machinery proteins and other regulatory proteins (such as SR and hnRNP), was fine-tuned by heat-stress priming to ensure proper responses and survival under transient, lethal high-temperature conditions (Palusa *et al.*, 2007; Reddy and Shad Ali, 2011; Yeap *et al.*, 2012; Palusa and Reddy, 2015). Co-transcriptional AS might be controlled epigenetically to produce the AS patterns needed for proper responses to ensure plant survival. In mammalian systems, it has been shown that chromatin organization involving epigenetic modifications and the rate of transcription regulate AS (Naftelberg *et al.*, 2015). The fact that the response to heat stress is transcriptionally regulated at the chromatin level (Kumar and Wigge, 2010; Strenkert *et al.*, 2011; Sawarkar and Paro, 2013) suggests that heat stress-induced AS patterns might be epigenetically controlled, a notion that requires further investigation. Heat stress is known to alter the localization of splicing regulators, which may also be responsible for observed changes in splicing patterns (Ali *et al.*, 2003).

Further studies are needed to explore how splicing memory is acquired and maintained in primed plants, and to identify the molecular mechanisms that underpin the interplay between heat stress, transcriptional regulation, epigenetics, and splicing memory. Under natural conditions, plants are faced with a variety of combined abiotic stress conditions. Our findings open up exciting possibilities for future research on the priming of single and combined stress factors, and further elucidation of the mechanisms that contribute to the induction of splicing memory by heat-stress priming might have practical applications for increasing abiotic stress tolerance.

## Supplementary data

Supplementary data are available at *JXB* online.

Fig. S1. Heatmaps showing changes in gene expression in response to heat stress.

Fig. S2. Functional categories of heat memory with interactions of heat-memory genes.

Fig. S3. Comparisons of DEGs between primed and non-primed plants after heat shock, and heat-responsive genes.

Fig. S4. Gene expression pattern of heat-priming memory-related DEGs and DIR-related genes.

Fig. S5. Differential AS events and genes between primed and non-primed plants.

Fig. S6. Splicing pattern of heat-responsive genes.

Fig. S7. IVG images of HSF genes.

Table S1. Annotated AS events.

Table S2. AS events and related genes at different time-points.

Table S3. List of genes with differential intron retention between various time-points.

Supplementary Figures and Tables

## References

[CIT0001] Ali GS, Golovkin M, Reddy AS 2003 Nuclear localization and *in vivo* dynamics of a plant-specific serine/arginine-rich protein. The Plant Journal 36, 883–893. 14675452 10.1046/j.1365-313x.2003.01932.x

[CIT0002] AlShareef S, Ling Y, Butt H, Mariappan KG, Benhamed M, Mahfouz MM 2017 Herboxidiene triggers splicing repression and abiotic stress responses in plants. BMC Genomics 18, 260. 28347276 10.1186/s12864-017-3656-z PMC5369228

[CIT0003] Avramova Z 2015 Transcriptional ‘memory’ of a stress: transient chromatin and memory (epigenetic) marks at stress-response genes. The Plant Journal 83, 149–159. 25788029 10.1111/tpj.12832

[CIT0004] Banti V, Mafessoni F, Loreti E, Alpi A, Perata P 2010 The heat-inducible transcription factor HsfA2 enhances anoxia tolerance in Arabidopsis. Plant Physiology 152, 1471–1483. 20089772 10.1104/pp.109.149815 PMC2832282

[CIT0005] Baurle I 2016 Plant heat adaptation: priming in response to heat stress. F1000Research 5, 694. 10.12688/f1000research.7526.1 PMC4837978 27134736

[CIT0006] Berry S, Dean C 2015 Environmental perception and epigenetic memory: mechanistic insight through FLC. The Plant Journal 83, 133–148. 25929799 10.1111/tpj.12869 PMC4691321

[CIT0007] Biamonti G, Caceres JF 2009 Cellular stress and RNA splicing. Trends in Biochemical Sciences 34, 146–153. 19208481 10.1016/j.tibs.2008.11.004

[CIT0008] Bita CE, Gerats T 2013 Plant tolerance to high temperature in a changing environment: scientific fundamentals and production of heat stress-tolerant crops. Frontiers in Plant Science 4, 273. 23914193 10.3389/fpls.2013.00273 PMC3728475

[CIT0009] Bond U 1988 Heat shock but not other stress inducers leads to the disruption of a sub-set of snRNPs and inhibition of *in vitro* splicing in HeLa cells. The EMBO Journal 7, 3509–3518. 2974799 10.1002/j.1460-2075.1988.tb03227.x PMC454852

[CIT0010] Boothby TC, Wolniak SM 2011 Masked mRNA is stored with aggregated nuclear speckles and its asymmetric redistribution requires a homolog of mago nashi. BMC Cell Biology 12, 45. 21995518 10.1186/1471-2121-12-45 PMC3205038

[CIT0011] Boothby TC, Zipper RS, van der Weele CM, Wolniak SM 2013 Removal of retained introns regulates translation in the rapidly developing gametophyte of *Marsilea vestita*. Developmental Cell 24, 517–529. 23434411 10.1016/j.devcel.2013.01.015

[CIT0012] Braunschweig U, Barbosa-Morais NL, Pan Q, Nachman EN, Alipanahi B, Gonatopoulos-Pournatzis T, Frey B, Irimia M, Blencowe BJ 2014 Widespread intron retention in mammals functionally tunes transcriptomes. Genome Research 24, 1774–1786. 25258385 10.1101/gr.177790.114 PMC4216919

[CIT0013] Brooks AN, Yang L, Duff MO, Hansen KD, Park JW, Dudoit S, Brenner SE, Graveley BR 2011 Conservation of an RNA regulatory map between *Drosophila* and mammals. Genome Research 21, 193–202. 20921232 10.1101/gr.108662.110 PMC3032923

[CIT0014] Bruce TJA, Matthes MC, Napier JA, Pickett JA 2007 Stressful “memories” of plants: Evidence and possible mechanisms. Plant Science 173, 603–608.

[CIT0015] Brzezinka K, Altmann S, Czesnick H et al 2016 Arabidopsis FORGETTER1 mediates stress-induced chromatin memory through nucleosome remodeling. eLIFE 5, e17061. 27680998 10.7554/eLife.17061 PMC5040591

[CIT0016] Burgess A, David R, Searle IR 2016 Deciphering the epitranscriptome: a green perspective. Journal of Integrative Plant Biology 58, 822–835. 27172004 10.1111/jipb.12483 PMC5094531

[CIT0017] Chang CY, Lin WD, Tu SL 2014 Genome-wide analysis of heat-sensitive alternative splicing in *Physcomitrella patens*. Plant Physiology 165, 826–840. 24777346 10.1104/pp.113.230540 PMC4044832

[CIT0018] Charng YY, Liu HC, Liu NY, Chi WT, Wang CN, Chang SH, Wang TT 2007 A heat-inducible transcription factor, HsfA2, is required for extension of acquired thermotolerance in Arabidopsis. Plant Physiology 143, 251–262. 17085506 10.1104/pp.106.091322 PMC1761974

[CIT0019] Charng YY, Liu HC, Liu NY, Hsu FC, Ko SS 2006 Arabidopsis Hsa32, a novel heat shock protein, is essential for acquired thermotolerance during long recovery after acclimation. Plant Physiology 140, 1297–1305. 16500991 10.1104/pp.105.074898 PMC1435801

[CIT0020] Chen M, Manley JL 2009 Mechanisms of alternative splicing regulation: insights from molecular and genomics approaches. Nature Reviews. Molecular Cell Biology 10, 741–754. 19773805 10.1038/nrm2777 PMC2958924

[CIT0021] Conrath U 2011 Molecular aspects of defence priming. Trends in Plant Science 16, 524–531. 21782492 10.1016/j.tplants.2011.06.004

[CIT0022] Cui P, Zhang S, Ding F, Ali S, Xiong L 2014 Dynamic regulation of genome-wide pre-mRNA splicing and stress tolerance by the Sm-like protein LSm5 in Arabidopsis. Genome Biology 15, R1. 24393432 10.1186/gb-2014-15-1-r1 PMC4053965

[CIT0023] Dennis G Jr, Sherman BT, Hosack DA, Yang J, Gao W, Lane HC, Lempicki RA 2003 DAVID: database for annotation, visualization, and integrated discovery. Genome Biology 4, P3. 12734009 PMC193660

[CIT0024] Diernfellner AC, Schafmeier T, Merrow MW, Brunner M 2005 Molecular mechanism of temperature sensing by the circadian clock of *Neurospora crassa*. Genes & Development 19, 1968–1973. 16107616 10.1101/gad.345905 PMC1199567

[CIT0025] Ding F, Cui P, Wang Z, Zhang S, Ali S, Xiong L 2014 Genome-wide analysis of alternative splicing of pre-mRNA under salt stress in Arabidopsis. BMC Genomics 15, 431. 24897929 10.1186/1471-2164-15-431 PMC4079960

[CIT0026] Ding Y, Fromm M, Avramova Z 2012 Multiple exposures to drought ‘train’ transcriptional responses in Arabidopsis. Nature Communications 3, 740. 10.1038/ncomms1732 22415831

[CIT0027] Filichkin S, Priest HD, Megraw M, Mockler TC 2015 Alternative splicing in plants: directing traffic at the crossroads of adaptation and environmental stress. Current Opinion in Plant Biology 24, 125–135. 25835141 10.1016/j.pbi.2015.02.008

[CIT0028] Filichkin SA, Priest HD, Givan SA, Shen R, Bryant DW, Fox SE, Wong WK, Mockler TC 2010 Genome-wide mapping of alternative splicing in *Arabidopsis thaliana*. Genome Research 20, 45–58. 19858364 10.1101/gr.093302.109 PMC2798830

[CIT0029] González-Schain N, Dreni L, Lawas LM, Galbiati M, Colombo L, Heuer S, Jagadish KS, Kater MM 2016 Genome-wide transcriptome analysis during anthesis reveals new insights into the molecular basis of heat stress responses in tolerant and sensitive rice varieties. Plant & Cell Physiology 57, 57–68. 26561535 10.1093/pcp/pcv174

[CIT0030] Haslbeck M, Vierling E 2015 A first line of stress defense: small heat shock proteins and their function in protein homeostasis. Journal of Molecular Biology 427, 1537–1548. 25681016 10.1016/j.jmb.2015.02.002 PMC4360138

[CIT0031] Hatfield JL, Prueger JH 2015 Temperature extremes: effect on plant growth and development. Weather and Climate Extremes 10, 4–10.

[CIT0032] Hilker M, Schwachtje J, Baier M et al 2016 Priming and memory of stress responses in organisms lacking a nervous system. Biological Reviews of the Cambridge Philosophical Society 91, 1118–1133. 26289992 10.1111/brv.12215

[CIT0033] Hu Z, Song N, Zheng M et al 2015 Histone acetyltransferase GCN5 is essential for heat stress-responsive gene activation and thermotolerance in Arabidopsis. The Plant Journal 84, 1178–1191. 26576681 10.1111/tpj.13076

[CIT0034] Iida K, Seki M, Sakurai T, Satou M, Akiyama K, Toyoda T, Konagaya A, Shinozaki K 2004 Genome-wide analysis of alternative pre-mRNA splicing in *Arabidopsis thaliana* based on full-length cDNA sequences. Nucleic Acids Research 32, 5096–5103. 15452276 10.1093/nar/gkh845 PMC521658

[CIT0035] Ikeda M, Mitsuda N, Ohme-Takagi M 2011 Arabidopsis HsfB1 and HsfB2b act as repressors of the expression of heat-inducible Hsfs but positively regulate the acquired thermotolerance. Plant Physiology 157, 1243–1254. 21908690 10.1104/pp.111.179036 PMC3252156

[CIT0036] Jaskiewicz M, Conrath U, Peterhänsel C 2011 Chromatin modification acts as a memory for systemic acquired resistance in the plant stress response. EMBO Reports 12, 50–55. 21132017 10.1038/embor.2010.186 PMC3024125

[CIT0037] Kumar SV, Wigge PA 2010 H2A.Z-containing nucleosomes mediate the thermosensory response in Arabidopsis. Cell 140, 136–147. 20079334 10.1016/j.cell.2009.11.006

[CIT0038] Lal SV, Brahma B, Gohain M et al 2015 Splice variants and seasonal expression of buffalo *HSF* genes. Cell Stress & Chaperones 20, 545–554. 25655489 10.1007/s12192-014-0563-y PMC4406941

[CIT0039] Laloum T, Martín G, Duque P 2018 Alternative splicing control of abiotic stress responses. Trends in Plant Science 23, 140–150. 29074233 10.1016/j.tplants.2017.09.019

[CIT0040] Lämke J, Brzezinka K, Altmann S, Bäurle I 2016a A hit-and-run heat shock factor governs sustained histone methylation and transcriptional stress memory. The EMBO Journal 35, 162–175. 26657708 10.15252/embj.201592593 PMC4718455

[CIT0041] Lämke J, Brzezinka K, Bäurle I 2016b HSFA2 orchestrates transcriptional dynamics after heat stress in *Arabidopsis thaliana*. Transcription 7, 111–114. 27383578 10.1080/21541264.2016.1187550 PMC4984677

[CIT0042] Larkindale J, Vierling E 2008 Core genome responses involved in acclimation to high temperature. Plant Physiology 146, 748–761. 18055584 10.1104/pp.107.112060 PMC2245833

[CIT0043] Lin MY, Chai KH, Ko SS, Kuang LY, Lur HS, Charng YY 2014 A positive feedback loop between HEAT SHOCK PROTEIN101 and HEAT STRESS-ASSOCIATED 32-KD PROTEIN modulates long-term acquired thermotolerance illustrating diverse heat stress responses in rice varieties. Plant Physiology 164, 2045–2053. 24520156 10.1104/pp.113.229609 PMC3982761

[CIT0044] Ling Y, Alshareef S, Butt H et al 2017 Pre-mRNA splicing repression triggers abiotic stress signaling in plants. The Plant Journal 89, 291–309. 27664942 10.1111/tpj.13383

[CIT0045] Liu HC, Charng YY 2012 Acquired thermotolerance independent of heat shock factor A1 (HsfA1), the master regulator of the heat stress response. Plant Signaling & Behavior 7, 547–550. 22516818 10.4161/psb.19803 PMC3419016

[CIT0046] Liu HC, Liao HT, Charng YY 2011 The role of class A1 heat shock factors (HSFA1s) in response to heat and other stresses in Arabidopsis. Plant, Cell & Environment 34, 738–751. 10.1111/j.1365-3040.2011.02278.x 21241330

[CIT0047] Liu J, Sun N, Liu M, Liu J, Du B, Wang X, Qi X 2013 An autoregulatory loop controlling Arabidopsis *HsfA2* expression: role of heat shock-induced alternative splicing. Plant Physiology 162, 512–521. 23503691 10.1104/pp.112.205864 PMC3641227

[CIT0048] Liu JG, Qin QL, Zhang Z, Peng RH, Xiong AS, Chen JM, Yao QH 2009 *OsHSF7* gene in rice, *Oryza sativa* L., encodes a transcription factor that functions as a high temperature receptive and responsive factor. BMB Reports 42, 16–21. 19192388 10.5483/bmbrep.2009.42.1.016

[CIT0049] Mauch-Mani B, Baccelli I, Luna E, Flors V 2017 Defense priming: an adaptive part of induced resistance. Annual Review of Plant Biology 68, 485–512. 10.1146/annurev-arplant-042916-041132 28226238

[CIT0050] Meiri D, Breiman A 2009 Arabidopsis ROF1 (FKBP62) modulates thermotolerance by interacting with HSP90.1 and affecting the accumulation of HsfA2-regulated sHSPs. The Plant Journal 59, 387–399. 19366428 10.1111/j.1365-313X.2009.03878.x

[CIT0051] Mittler R, Finka A, Goloubinoff P 2012 How do plants feel the heat?Trends in Biochemical Sciences 37, 118–125. 22236506 10.1016/j.tibs.2011.11.007

[CIT0052] Morimoto RI, Santoro MG 1998 Stress-inducible responses and heat shock proteins: new pharmacologic targets for cytoprotection. Nature Biotechnology 16, 833–838. 10.1038/nbt0998-833 9743115

[CIT0053] Naftelberg S, Schor IE, Ast G, Kornblihtt AR 2015 Regulation of alternative splicing through coupling with transcription and chromatin structure. Annual Review of Biochemistry 84, 165–198. 10.1146/annurev-biochem-060614-034242 26034889

[CIT0054] Nishizawa-Yokoi A, Nosaka R, Hayashi H et al 2011 HsfA1d and HsfA1e involved in the transcriptional regulation of HsfA2 function as key regulators for the Hsf signaling network in response to environmental stress. Plant & Cell Physiology 52, 933–945. 21471117 10.1093/pcp/pcr045

[CIT0055] Palusa SG, Ali GS, Reddy AS 2007 Alternative splicing of pre-mRNAs of Arabidopsis serine/arginine-rich proteins: regulation by hormones and stresses. The Plant Journal 49, 1091–1107. 17319848 10.1111/j.1365-313X.2006.03020.x

[CIT0056] Palusa SG, Reddy AS 2015 Differential recruitment of splice variants from SR pre-mRNAs to polysomes during development and in response to stresses. Plant & Cell Physiology 56, 421–427. 25637375 10.1093/pcp/pcv010

[CIT0057] **Prime-A-Plant Group** 2006 Priming: getting ready for battle. Molecular Plant-Microbe Interactions 19, 1062–1071. 17022170 10.1094/MPMI-19-1062

[CIT0058] Reddy AS, Marquez Y, Kalyna M, Barta A 2013 Complexity of the alternative splicing landscape in plants. The Plant Cell 25, 3657–3683. 24179125 10.1105/tpc.113.117523 PMC3877793

[CIT0059] Reddy AS, Shad Ali G 2011 Plant serine/arginine-rich proteins: roles in precursor messenger RNA splicing, plant development, and stress responses. WIREs RNA 2, 875–889. 21766458 10.1002/wrna.98

[CIT0060] Richter K, Haslbeck M, Buchner J 2010 The heat shock response: life on the verge of death. Molecular Cell 40, 253–266. 20965420 10.1016/j.molcel.2010.10.006

[CIT0061] Sani E, Herzyk P, Perrella G, Colot V, Amtmann A 2013 Hyperosmotic priming of Arabidopsis seedlings establishes a long-term somatic memory accompanied by specific changes of the epigenome. Genome Biology 14, R59. 23767915 10.1186/gb-2013-14-6-r59 PMC3707022

[CIT0062] Sawarkar R, Paro R 2013 Hsp90@chromatin.nucleus: an emerging hub of a networker. Trends in Cell Biology 23, 193–201. 23286900 10.1016/j.tcb.2012.11.007

[CIT0063] Scharf KD, Berberich T, Ebersberger I, Nover L 2012 The plant heat stress transcription factor (Hsf) family: structure, function and evolution. Biochimica et Biophysica Acta 1819, 104–119. 22033015 10.1016/j.bbagrm.2011.10.002

[CIT0064] Sedaghatmehr M, Mueller-Roeber B, Balazadeh S 2016 The plastid metalloprotease FtsH6 and small heat shock protein HSP21 jointly regulate thermomemory in Arabidopsis. Nature Communications 7, 12439. 10.1038/ncomms12439 PMC5007455 27561243

[CIT0065] Shalgi R, Hurt JA, Lindquist S, Burge CB 2014 Widespread inhibition of posttranscriptional splicing shapes the cellular transcriptome following heat shock. Cell Reports 7, 1362–1370. 24857664 10.1016/j.celrep.2014.04.044

[CIT0066] Staiger D, Brown JW 2013 Alternative splicing at the intersection of biological timing, development, and stress responses. The Plant Cell 25, 3640–3656. 24179132 10.1105/tpc.113.113803 PMC3877812

[CIT0067] Stief A, Brzezinka K, Lämke J, Bäurle I 2014 Epigenetic responses to heat stress at different time scales and the involvement of small RNAs. Plant Signaling & Behavior 9, e970430. 25482804 10.4161/15592316.2014.970430 PMC4622961

[CIT0068] Strenkert D, Schmollinger S, Sommer F, Schulz-Raffelt M, Schroda M 2011 Transcription factor-dependent chromatin remodeling at heat shock and copper-responsive promoters in *Chlamydomonas reinhardtii*. The Plant Cell 23, 2285–2301. 21705643 10.1105/tpc.111.085266 PMC3160021

[CIT0069] Sugio A, Dreos R, Aparicio F, Maule AJ 2009 The cytosolic protein response as a subcomponent of the wider heat shock response in Arabidopsis. The Plant Cell 21, 642–654. 19244141 10.1105/tpc.108.062596 PMC2660624

[CIT0070] Sun W, Bernard C, van de Cotte B, Van Montagu M, Verbruggen N 2001 At-HSP17.6A, encoding a small heat-shock protein in Arabidopsis, can enhance osmotolerance upon overexpression. The Plant Journal 27, 407–415. 11576425 10.1046/j.1365-313x.2001.01107.x

[CIT0071] Swindell WR, Huebner M, Weber AP 2007 Plastic and adaptive gene expression patterns associated with temperature stress in *Arabidopsis thaliana*. Heredity 99, 143–150. 17473866 10.1038/sj.hdy.6800975

[CIT0072] Tang T, Yu A, Li P, Yang H, Liu G, Liu L 2016 Sequence analysis of the Hsp70 family in moss and evaluation of their functions in abiotic stress responses. Scientific Reports 6, 33650. 27644410 10.1038/srep33650 PMC5028893

[CIT0073] Trapnell C, Pachter L, Salzberg SL 2009 TopHat: discovering splice junctions with RNA-Seq. Bioinformatics 25, 1105–1111. 19289445 10.1093/bioinformatics/btp120 PMC2672628

[CIT0074] Wang Z, Ji H, Yuan B, Wang S, Su C, Yao B, Zhao H, Li X 2015 ABA signalling is fine-tuned by antagonistic HAB1 variants. Nature Communications 6, 8138. 10.1038/ncomms9138 26419884

[CIT0075] Weng M, Yang Y, Feng H, Pan Z, Shen WH, Zhu Y, Dong A 2014 Histone chaperone ASF1 is involved in gene transcription activation in response to heat stress in *Arabidopsis thaliana*. Plant, Cell & Environment 37, 2128–2138. 10.1111/pce.12299 24548003

[CIT0076] Wu TY, Juan YT, Hsu YH, Wu SH, Liao HT, Fung RW, Charng YY 2013 Interplay between heat shock proteins HSP101 and HSA32 prolongs heat acclimation memory posttranscriptionally in Arabidopsis. Plant Physiology 161, 2075–2084. 23439916 10.1104/pp.112.212589 PMC3613477

[CIT0077] Yan K, Liu P, Wu CA, Yang GD, Xu R, Guo QH, Huang JG, Zheng CC 2012 Stress-induced alternative splicing provides a mechanism for the regulation of microRNA processing in *Arabidopsis thaliana*. Molecular Cell 48, 521–531. 23063528 10.1016/j.molcel.2012.08.032

[CIT0078] Yeap WC, Ooi TE, Namasivayam P, Kulaveerasingam H, Ho CL 2012 *EgRBP42* encoding an hnRNP-like RNA-binding protein from *Elaeis guineensis* Jacq. is responsive to abiotic stresses. Plant Cell Reports 31, 1829–1843. 22699852 10.1007/s00299-012-1297-x

[CIT0079] Zhang JX, Wang C, Yang CY, Wang JY, Chen L, Bao XM, Zhao YX, Zhang H, Liu J 2010 The role of Arabidopsis AtFes1A in cytosolic Hsp70 stability and abiotic stress tolerance. The Plant Journal 62, 539–548. 20536787 10.1111/j.1365-313X.2010.04173.x

