# Supplementary material for: Thermopriming triggers splicing memory in Arabidopsis
Source: J Exp Bot. 2018 Feb 27;69(10):2659–75. doi: 10.1093/jxb/ery062 (PMC5920379; doi:10.1093/jxb/ery062)
Supplement: Supplementary Figures and Tables [file ery062_suppl_supplementary_figures-and-tables.pdf]

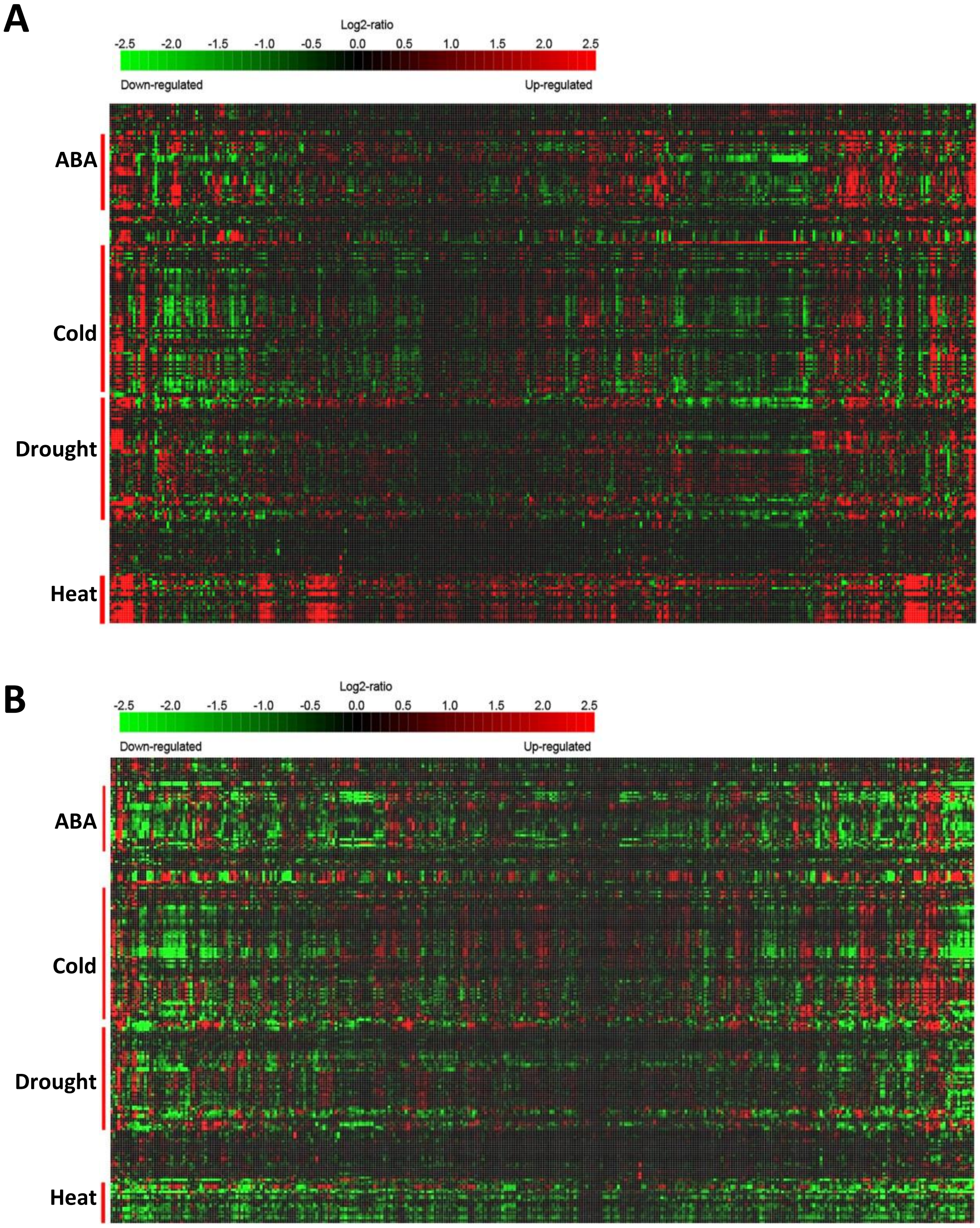

Supplementary Figure 1. Gene expression Changed by heat stress in our study correspond to earlier studies. The heatmaps were generated by mapping the heat responsive genes defined by RNA-seq data to the microarray database using Genevestigator. The heatmaps indicate that the heat responsive genes are consistent with previous microarray experiment. A, up-regulated genes. B, down-regulated genes.

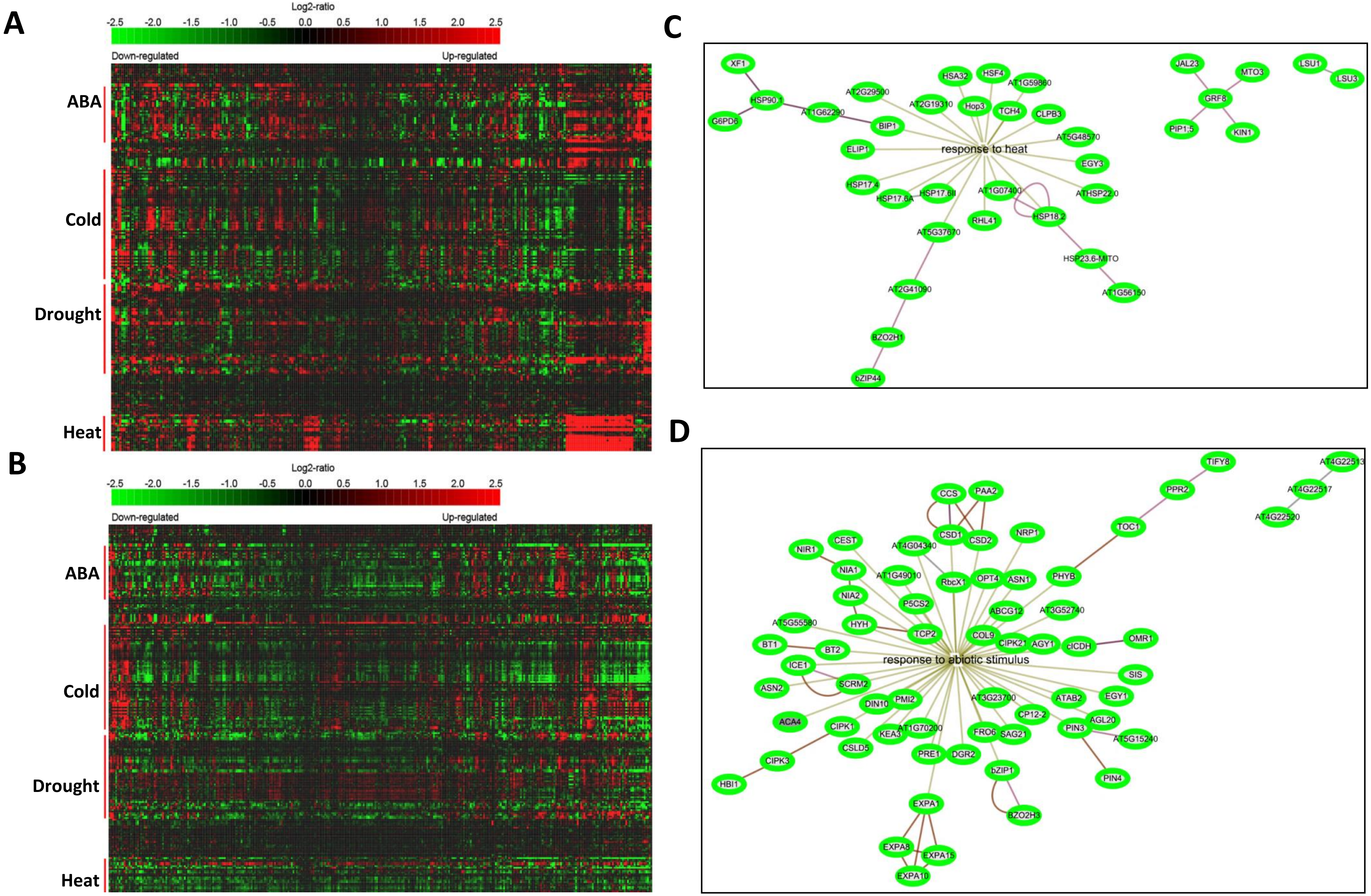

Supplementary Figure 2. Functional category of Heat memory. A and B, The heatmaps indicate that the Heat memory genes are associated with heat treatment. A and B show the up and down regulated genes, respectively. C and D, The interaction network of the heat memory genes. C, The interaction network of the heat memory genes in response to heat. D, The interaction network of the heat memory genes in response to abiotic stress.

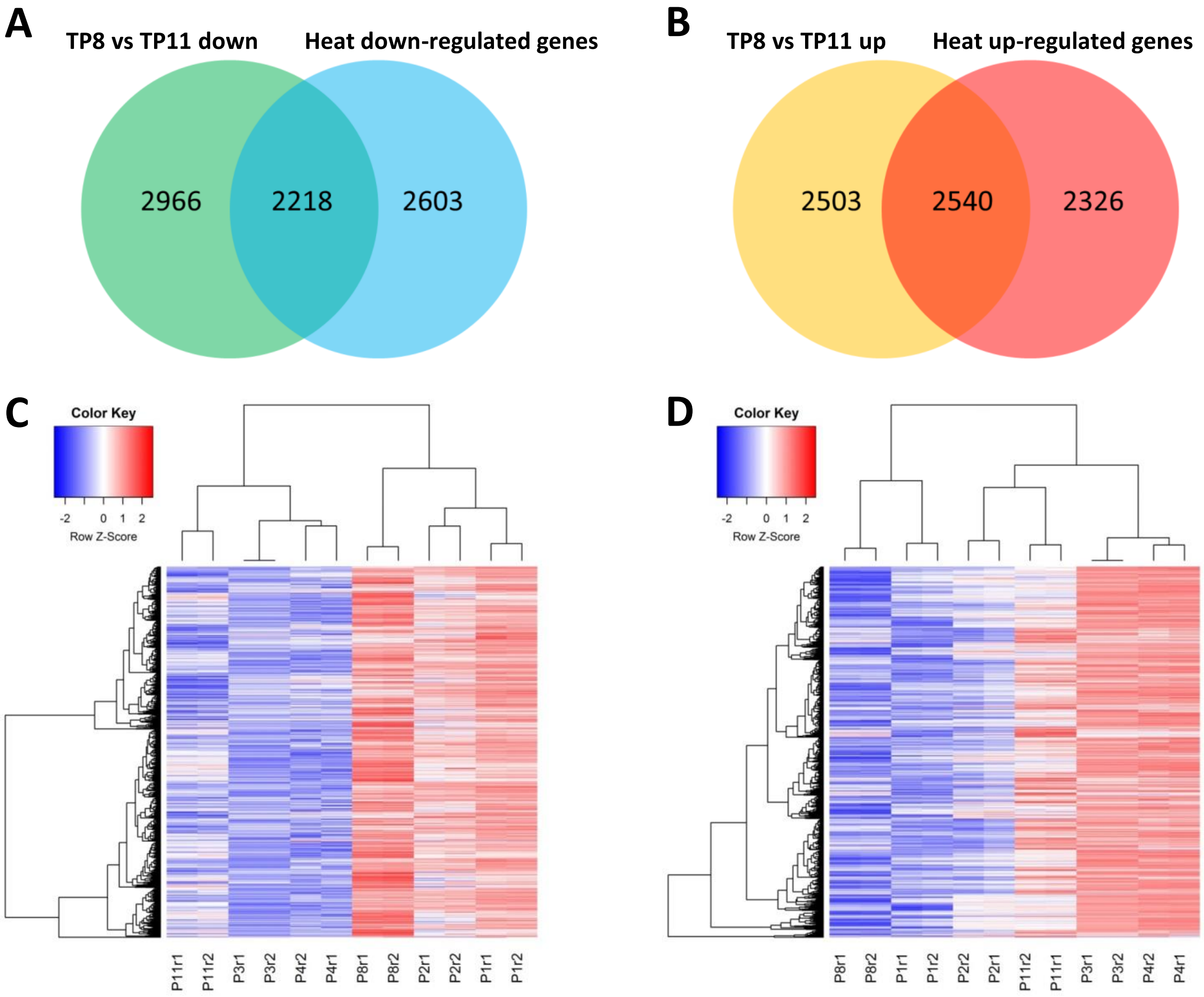

Supplementary Figure 3. Comparisons of the DEGs between primed vs non-primed plants in 2d after heat shock (TP8 vs TP11) and heat-responsive genes (TP2TP3TP4 vs TP1). A and B show down-regulated (A) and up-regulated (B) genes in TP11 overlapping to heat-responsive genes, respectively. C and D, The clustering of expression profiles of the heat responsive genes whose expression can not be returned in non-primed plants after heat shock (TP11). C, heat down-regulated genes, D, heat up-regulated genes.

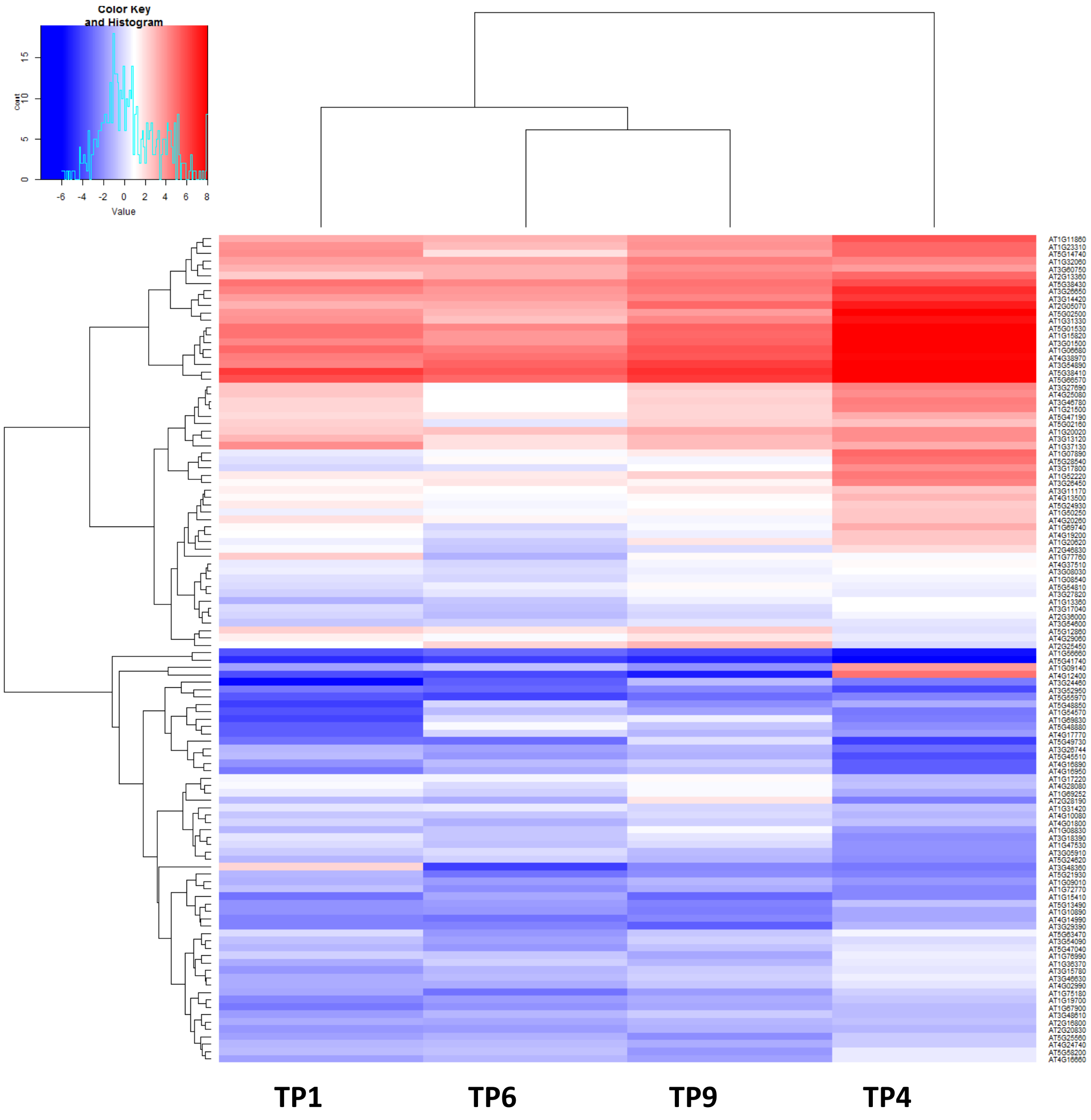

Supplementary Figure 4. Gene expression pattern of heat priming memory-related DEGs and DIR related genes. Expression patterns of 124 genes which are differentially express and also possess differentially expressed intron(s) in both heat priming phases (TP4) and recovery phases (TP6).

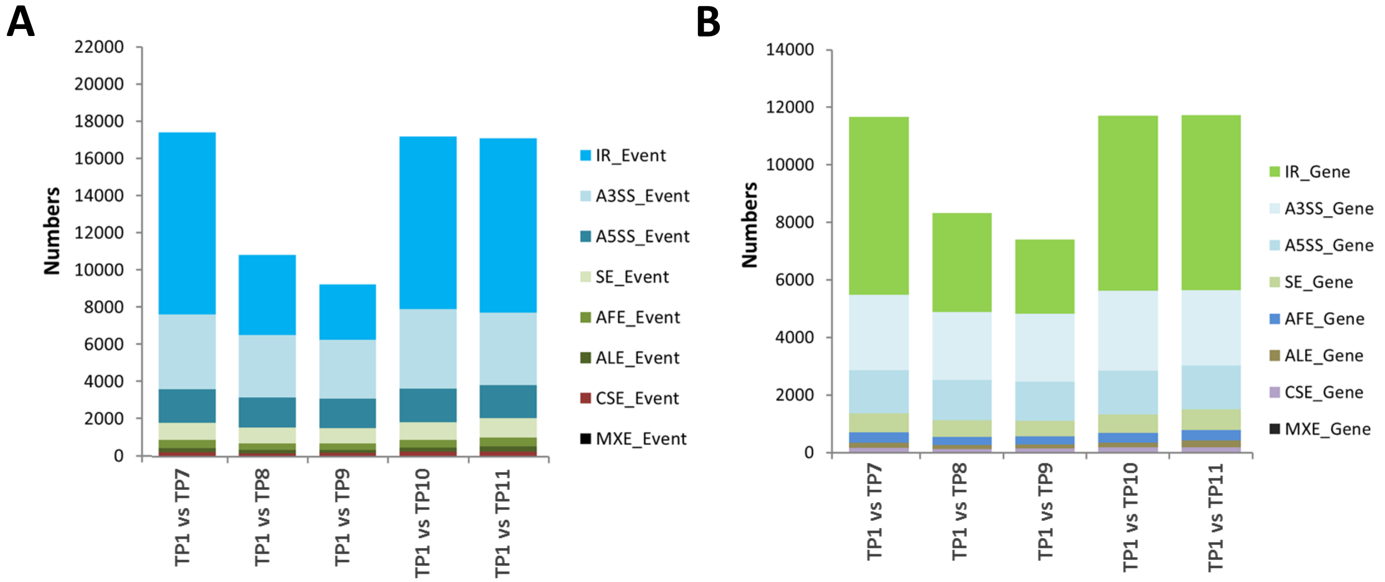

Supplementary Figure 5. Differential AS events and genes between primed and non-primed plants. A, Column plots show the number of differential AS events from primed and non-primed plants under lethal heat shock and recovery 2d. B, Column plots show numbers of genes that contain differential AS events from primed and non-primed plants under lethal heat shock and recovery 2d. During lethal heat shock phase, the numbers of differential AS events / -related genes are similar between primed and non-primed plants (see TP1 vs TP7 and TP1 vs TP10 in A and B), and the numbers of differential AS events / -related genes in primed plants drop down closed to control level (see TP1 vs TP8, TP1 vs TP7 and TP1 vs TP9 in A and B), whereas the number in non-primed plant stay the same as that of heat shock plants (see TP1 vs TP10, TP1 vs TP9 and TP1 vs TP11 in A and B).

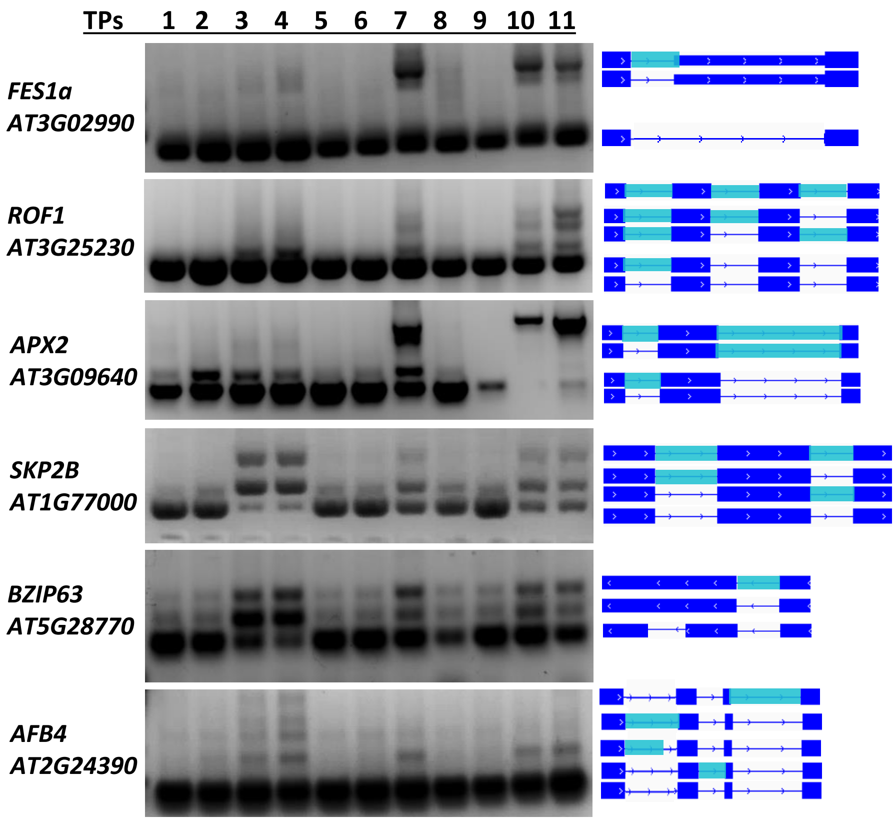

Supplementary Figure 6. splicing pattern of heat-responsive genes. RT-PCR was performed using primers that flank introns. One or more isoforms of genes are expressed at different time points (TPs). The gene structure flanking the amplified fragments and the structures of regulated variants are shown beside the gel images. Blue box, exon; narrow blue box, 3' or 5' UTR; line, spliced intron; light blue, retained intron. These genes tend to express more intron retained isoforms in TP 3, TP4, TP7, TP10 and TP11. Genes have different ratio of intron retention in heat priming and heat shock. *FES1a*, *ROF1* and *APX2* expressed less intron retained isoforms in heat priming (TP 3 and TP4), when compared to that of heat shock (TP 7 and TP10). *SKP2B*, *BZIP63* and *AFB4* expressed more intron retained isoforms in heat priming, when compared to that of heat shock.

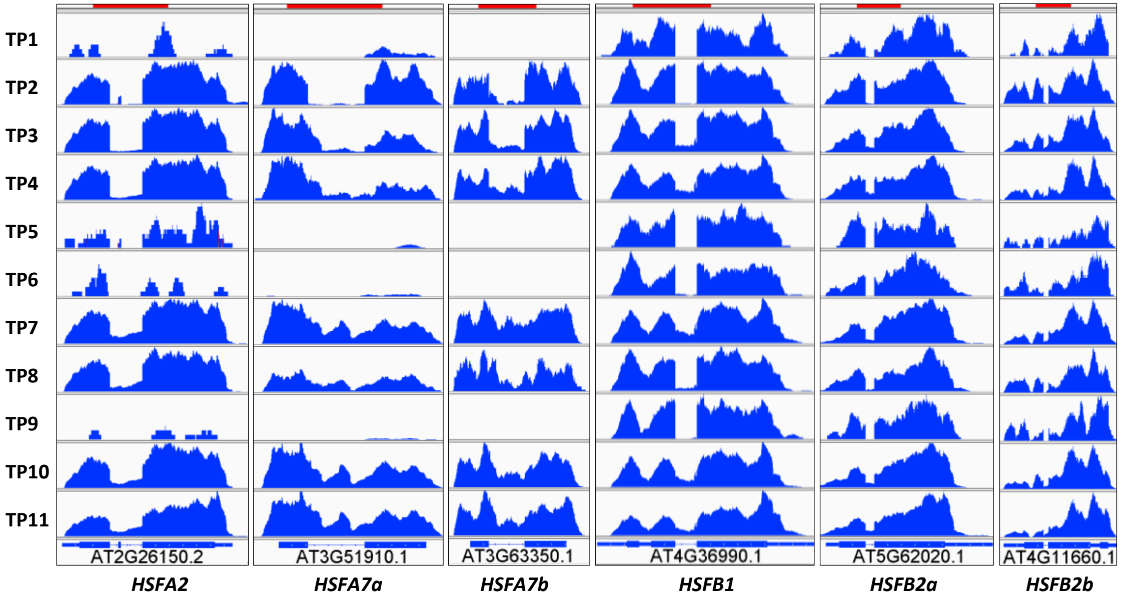

Supplementary Figure 7. IGV images of HSF genes. The gene structure and intron retention of interesting regions from these 6 HSF genes are shown from the IGV program. Coverage of RT-PCR amplified fragments are shown on the top of each panel (red bar).

Supplementary Table 1: The annotated AS events

Type	IR	A3SS	A5SS	SE	AFE	ALE	CSE	MXE
TAIR10.gff	127555	2243	1220	739	679	323	102	18
TAIR10.gff+novel junctions	127555	19577	6574	5264	2173	843	956	19
Events	38131	12319	5535	3655	2103	771	678	18

IR: intron retention, A3SS: alternative 3' splice sites, A5SS: alternative 5' splice sites, SE, skipping exon, AFE: alternative first exons, ALE: alternative last exons, CSE: coordinated skipping exons, MXE: mutually exclusive exons.

Supplementary Table 2: AS events and related genes in different time points

Samples	AS events	Related genes	Intron-containing genes (%)
TP1	32724	11817	53.33
TP2	28277	10775	45.52
TP3	35138	11095	50.13
TP4	31825	10357	46.8
TP5	30304	11275	50.95
TP6	31892	11679	52.77
TP7	37318	11967	54.07
TP8	35995	12262	55.41
TP9	32306	11783	53.24
TP10	38629	12139	54.85
TP11	36676	11981	54.14

Supplementary Table 3: The list of genes contain differential intron retention between TP2, 3 , 4 and 6, compared to TP1 and 9

Genes with more intron in TP2, 3, 4, and 6, compared to TP1 and 9	Genes with less intron in TP2, 3, 4, and 6, compared to TP1 and 9
AT5G04550	AT2G36000
AT4G25080	AT4G31877
AT5G45510	AT5G02500
AT4G10730	AT2G32480
AT1G76490	AT1G69252
AT4G31550	AT1G22140
AT4G02500	AT1G78865
AT5G49730	AT3G16400
AT4G20940	
AT1G53910	
AT1G61180	
AT1G77460	
AT5G08335	
AT4G31550	
AT5G25280	
AT2G45340	
AT4G39090	
AT3G52800	
AT1G13190	
AT3G56860	
AT1G63880	
AT5G50960	
AT3G16000	
AT5G16110	
AT4G01950	
AT1G08540	
AT1G37130	
AT1G53910	
